# Plasma Membrane-Associated Proteins Identified in Arabidopsis Wild Type, *lbr2-2* and *bak1-4* Mutants Treated with LPSs from *Pseudomonas* *syringae* and *Xanthomonas campestris*

**DOI:** 10.3390/membranes12060606

**Published:** 2022-06-10

**Authors:** Benedict C. Offor, Msizi I. Mhlongo, Ian A. Dubery, Lizelle A. Piater

**Affiliations:** Department of Biochemistry, University of Johannesburg, Auckland Park, Johannesburg 2006, South Africa; benedictoffor@gmail.com (B.C.O.); mmhlongo@uj.ac.za (M.I.M.); idubery@uj.ac.za (I.A.D.)

**Keywords:** *Arabidopsis thaliana*, BAK1, LC-MS/MS, LBR2, LPS, MAMPs, PRRs, *Pseudomonas syringae*, *Xanthomonas campestris*

## Abstract

Plants recognise bacterial microbe-associated molecular patterns (MAMPs) from the environment via plasma membrane (PM)-localised pattern recognition receptor(s) (PRRs). Lipopolysaccharides (LPSs) are known as MAMPs from gram-negative bacteria that are most likely recognised by PRRs and trigger defence responses in plants. The Arabidopsis PRR(s) and/or co-receptor(s) complex for LPS and the associated defence signalling remains elusive. As such, proteomic identification of LPS receptors and/or co-receptor complexes will help to elucidate the molecular mechanisms that underly LPS perception and defence signalling in plants. The Arabidopsis LPS-binding protein (LBP) and bactericidal/permeability-increasing protein (BPI)-related-2 (LBR2) have been shown to recognise LPS and trigger defence responses while brassinosteroid insensitive 1 (BRI1)-associated receptor kinase 1 (BAK1) acts as a co-receptor for several PRRs. In this study, Arabidopsis wild type (WT) and T-DNA knock out mutants (*lbr2-2* and *bak1-4*) were treated with LPS chemotypes from *Pseudomonas syringae* pv. *tomato* DC3000 (*Pst*) and *Xanthomonas campestris* pv. *campestris* 8004 (*Xcc*) over a 24 h period. The PM-associated protein fractions were separated by liquid chromatography and analysed by tandem mass spectrometry (LC-MS/MS) followed by data analysis using Byonic^TM^ software. Using Gene Ontology (GO) for molecular function and biological processes, significant LPS-responsive proteins were grouped according to defence and stress response, perception and signalling, membrane transport and trafficking, metabolic processes and others. Venn diagrams demarcated the MAMP-responsive proteins that were common and distinct to the WT and mutant lines following treatment with the two LPS chemotypes, suggesting contributions from differential LPS sub-structural moieties and involvement of LBR2 and BAK1 in the LPS-induced MAMP-triggered immunity (MTI). Moreover, the identification of RLKs and RLPs that participate in other bacterial and fungal MAMP signalling proposes the involvement of more than one receptor and/or co-receptor for LPS perception as well as signalling in Arabidopsis defence responses.

## 1. Introduction

Plants perceive microbe-associated molecular patterns (MAMPs) via pattern recognition receptor(s) (PRRs) and trigger defence responses such as reactive oxygen species (ROS) production, callose deposition, mitogen-activated protein kinase (MAPK) activation, expression of pathogenesis-related (PR) proteins and accumulation defence-related secondary metabolites [1,2,3]. Gram-negative bacterial LPSs are MAMPs that trigger defence responses or prime a plant to respond more rapidly to subsequent pathogen attacks [4]. The archetypal LPS structure is composed of three sub-components that include the lipid A, core oligosaccharides and O-polysaccharide (OPS) that can differ within and across species [5,6]. Studies have shown that LPS and its sub-components are capable of inducing defence responses in plants, with the most conserved lipid A shown to be the major determinant of the immunostimulatory capacity [6,7,8,9]. The OPS chain contains sugar repeating units, which determine whether the LPS is ‘smooth’ (when the repeating oligosaccharide is intact) or ‘rough’ (when the repeating motif is shortened or absent) [10]. The conserved 2-keto-3-deoxyoctanoic acid (Kdo) links lipid A to the core oligosaccharide (inner and outer core) [6]. The acylation pattern of the lipid A moiety of the LPS contributes significantly to its variability and endotoxicity [11]. In this regard, the lipid A moiety of the LPS from *Xanthomonas campestris* pv. *campestris* 8004 (*Xcc*) is hexa-acylated, while that of *Pseudomonas syringae* pv. *tomato* DC3000 (*Pst*) is penta-/hexa-acylated [12,13,14].

The LPS perception system in mammals is well studied, but little is known about its recognition and signalling in plants. In mammals, LPS is recognised by the LPS binding protein (LBP), which transfers it to a complex that comprises soluble myeloid differentiation protein-2 (MD-2), cluster of differentiation 14 (CD14) and Toll-like receptor 4 (TLR4), thereby triggering defence responses [15,16,17]. In addition, studies have reported a TLR4-independent cytosolic LPS binding and the induction of pyroptosis through human caspase-4, the mouse caspase homologue-11 and also human caspase-5 [18,19]. In Arabidopsis, a membrane-spanning receptor-like kinase (RLK) (lipooligosaccharide-specific reduced elicitation—LORE) was thought to be a PRR for LPS but was later resolved to recognise a medium-chain 3-hydroxy fatty acid (mc-3-OH-FAs) as a MAMP and trigger defence responses in Arabidopsis [20,21,22]. LBP/BPI related-1 (AtLBR-1) and LBP/BPI related-2 (AtLBR-2) have been shown to perceive LPS from *Pseudomonas aeruginosa* and trigger defence responses in Arabidopsis [23]. Recently, Hussan et al. [24] identified LBR1 as an *Xcc* LPS-responsive PM-associated protein in Arabidopsis.

Several PRRs and their cognate MAMPs have been characterised in plants. For instance, flagellin-sensing 2 (FLS2) and elongation factor Tu receptor (EFR) recognise the bacterial epitopes of the N-terminus flagellin peptides (flg22) and elongation factor-thermo unstable (EF-Tu), respectively, and trigger defence responses in Arabidopsis [25,26]. To enhance defence responses against invading pathogens, some of these PRRs (e.g., FLS2) interact with co-receptors, such as brassinosteroid insensitive 1 (BRI1)-associated receptor kinase (BAK1), upon ligand recognition [27]. BAK1 is a well-studied multifunctional co-receptor to several receptor proteins, including those involved in brassinosteroid—MAMP—and damage-associated molecular pattern (DAMP) signalling [28]. To avoid autoimmunity, BAK1 is only released from the complex between BAK1 and the BAK1-interacting receptor-like kinase 2 (BIR2) to form another complex with FLS2 in a flg22-dependent manner [29]. Previously, BAK1 was identified as one of the LPS-responsive proteins in Arabidopsis [30,31].

The plasma membrane (PM) regulates cell external and internal communication with the aid of the proteins embedded in the membrane phospholipid bilayer, which plays a key role in the initial perception of pathogen-derived signals [32,33]. In addition, membrane rafts within the PM regulate several physiochemical processes, such as transport, trafficking and signalling, and are potential hubs for specific receptor (e.g., FLS2) and co-receptor (e.g., BAK1) complex assembly involved in biotic/abiotic stress defence responses [2,34,35].

PRRs are either RLKs with ligand-binding external ectodomains, a transmembrane domain and internal kinase domain, or RLPs that lack the kinase domain [36]. These RLKs or RLPs can be classified based on the motifs or domains of their ectodomains, which include leucine-rich repeat domain (LRR), lysin M (LysM), lectin (Lec) or epidermal growth factor (EGF)-like domains [3]. For example, FLS2 (bacterial flg22 receptor), CERK1 (fungal chitin receptor) and LORE (bacterial mc-3-OH-FAs receptor) are LRR-RLK, LysM-RLK and Lec-RLK proteins, respectively [21,37,38]. The receptor-like cytoplasmic kinases (RLCKs) cannot recognise MAMPs as they lack ectodomains and transmembrane domains but have kinase domains for the activation of the downstream proteins involved in defence signalling [36]. For instance, a RLCK Botrytis-induced kinase1 (BIK1) plays a vital role downstream of PRRs where it is involved in the transduction of MAMP signals from the receptor complex to downstream intracellular signalling, including activation of RbohD-mediated ROS production [39,40].

Pathogens use several mechanisms to suppress innate immunity in plants and intensify disease severity. For instance, the *P. syringae* effector HopAO1 is reported to target LORE and disrupt its ability to phosphorylate and activate the receptor-like cytoplasmic kinase (RLCK) PBL34, thereby suppressing immune responses [22]. Additionally, upon flg22 treatment, FLS2 undergoes ubiquitin-mediated proteasome degradation mediated by U-box E3 ubiquitin ligases (PUB12 and PUB13) and attenuates immune signalling in Arabidopsis [41]. On the other hand, plants counter this effector-triggered susceptibility (ETS) using the second layer of defence, termed effector-triggered immunity (ETI), which is characterised by the hypersensitive response (HR). ETI is induced via the recognition of avirulent pathogenic effectors by plant resistance (R) proteins, termed nucleotide-binding site leucine-rich repeat receptor-like kinase (NB-LRR-RLK) proteins [36,42].

Understanding the LPS PRRs and/or co-receptors in plants will uncover its recognition (and/or moieties involved in recognition) and associated defence signalling. Here, LPS chemotypes from *Pst* and *Xcc* (LPS*_Pst_* and LPS*_Xcc_*) were used to treat Arabidopsis WT and mutants (*lbr2-2* and *bak1-4*) for a 24 h period. Using label-free LC-MS/MS proteomics, distinct LPS-responsive proteins, including those that participate in perception and signalling as well as in defence and stress responses, were identified in WT and mutants. This study afforded new insights into the mechanism of the perception of different LPS chemotypes by Arabidopsis, and thus may contribute to ongoing research on developing plants that are more resistant to pathogens and possibly improve global food security.

## 2. Materials and Methods

### 2.1. LPS Isolation and Characterisation

LPS was isolated and characterised for suitability of downstream experimentation according to Tinte et al. [43]. In this regard LPSs from both *Pst* and *Xcc* were included following purification using the hot phenol-water method [44]. Lyophilised bacterial cells (20 g) were ground in liquid nitrogen with a mortar and pestle and suspended in equal volumes of hot distilled water (dH_2_O) and 90% (*w*/*v*) phenol (Merck, Gauteng, SA) at 65–70 °C (final concentration of dry cells was 2.5% (*w*/*v*)). The mixture was allowed to stir at 65—0 °C for 30 min, and then cooled on ice to 10 °C to facilitate phase separation during centrifugation at 8500× *g*, 4 °C for 30 min. The upper water phase was collected, and the extraction was repeated three times with the lower phenol phase and the insoluble interphase to improve LPS yield. The resultant water phases were pooled and dialysed against dH_2_O using 7000 MWCO SnakeSkin^®^ Dialysis Tubing (ThermoFisher Scientific, Rockford, IL, USA) with 4 changes of dH_2_O for 4 d to remove phenol and small molecular weight bacterial substances. The dialysed solution was centrifuged at 8500× *g*, 4 °C for 20 min, and the supernatant lyophilised. To further purify the LPS from co-extracted contaminants, the extract was re-suspended in 20 mL dH_2_O followed by the addition of 0.05 mg of deoxyribonuclease (DNase; Roche Diagnostics, Mannheim, Germany), ribonuclease (RNase; Roche Diagnostics, Mannheim, Germany) and Proteinase K (Merck, Darmstadt, Germany), and the solution incubated at 37 °C for 2 h. An equal volume (20 mL) of 90% (*w*/*v*) phenol at 65–70 °C was added to the solution, vortexed and centrifuged at 8500× *g*, 4 °C for 20 min. The upper water phase was collected and dialysed against dH_2_O for 4 d with multiple changes of dH_2_O. After dialysis, LPS was lyophilised and stored at −20 °C.

### 2.2. Plant Growth Conditions and Genotyping

For evaluating the homozygosity of mutant lines, the SALK guidelines were followed. In this regard, wild type (WT) *Arabidopsis thaliana* (ecotype Columbia, Col-0) and T-DNA insertion mutant *lbr2-2* (At3g20270, SALK_132326) were obtained from Arabidopsis Biological Resource Center (ABRC) (The Ohio State University, OH, USA). Another mutant *bak1-4* (At4g33430, SALK_116202) was a gift from Professor Thorsten Nürnberger (University of Tübingen, Tübingen, Germany). The SALK guidelines included a two-step PCR genotyping assay [45] to ascertain mutant plants that were homozygous for T-DNA inserts using primer design tools of the Salk Institute Genome Analysis Laboratory (SIGnAL) [46]. Primers used include: Atlbr2-2-LP: 5′-CCTCTCAGAAAGATGCATTGG-3′; Atlbr2-2-RP: 5′-CCCTGTCTTAGGGAATTCGTC-3′; Atbak1-4-LP: 5′-CATGACATCATCATCATTCGC-3′; Atbak1-4-RP: 5′-ATTTTGCAGTTTTGCCAACAC-3′; T-DNA primer LBb1.3: 5′-ATTTTGCCGATTTCGGGAAC-3′.

Arabidopsis plants (WT and mutants) were grown in a controlled plant room for 8 weeks as described in Hussan et al. [24] and used for the experiments.

### 2.3. Plant Treatment and Harvesting

Arabidopsis WT and mutants (*lbr2-2* and *bak1-4*) were treated with LPSs (100 µg/mL) from *Pst* and *Xcc* by gentle pressure infiltration of the leaves as demonstrated in Hussan et al. [24]. For each time point, nine Arabidopsis plants were treated. Negative control plants were treated with sterile 2.5 mM MgCl_2_ (used to dissolve the LPSs) to account for any responses that may be generated thereby and wounding upon treatment [43,47]. The experimental design included leaves that were immediately harvested after 0, 12, 18, and 24 h, snap frozen in liquid nitrogen and stored at −80 °C until use.

### 2.4. Plasma Membrane (PM)-Associated Fraction Preparation

The PM-associated isolation was performed following the small-scale procedure as described by Giannini et al. [48] and Abas and Luschnig [49] with minor modifications. Here, we were not concerned with isolating pure PM fractions so as to also include PM-associated proteins that may be in complex with the PM and possibly participating in LPS-induced defence signalling. Arabidopsis leaves (10 g) were ground in liquid nitrogen with a pre-chilled mortar and pestle. The ground tissue was transferred into a beaker containing ice-cold 50 mL homogenising buffer (250 mM sucrose (Sigma-Aldrich, St. Louis, MO, USA), 3 mM EDTA (Saarchem, Gauteng, South Africa), 10% (*v*/*v*) glycerol, 250 mM potassium iodide (KI; Saarchem, Gauteng, South Africa), 2 mM phenylmethylsulfonyl fluoride (PMSF; Boehringer, Mannheim, Germany), 0.5% poly(vinylpolypyrrolidone) (PVPP; Sigma-Aldrich, St. Louis, MO, USA), 15 mM β-mercaptoethanol, 70 mM Tris-HCl pH 7.5, 4 mM dithiothreitol (DTT, ThermoFisher Scientific, Vilnius, Lithuania) and 1% bovine serum albumin (BSA, Boehringer, Mannheim, Germany)), and homogenised further with an Ultraturrax homogeniser (IKA, Staufen, Germany) for 2 min. The homogenate (HM) was filtered through a two-layered miracloth membrane (EMD Millipore, Burlington, MA, USA). The HM fraction was centrifuged at 6000× *g*, 4 °C for 3 min, in a fixed angle Beckman Avanti™ J-30 centrifuge. The pellet was discarded and the supernatant further centrifuged at 13,000× *g*, 4 °C for 25 min. The resultant supernatant was discarded and the pellet enriched with the microsomal fraction (MF) was collected and re-suspended with ice-cold MF buffer (250 mM sucrose, 10% (*v*/*v*) glycerol, 1 mM DTT, 1 mM PMSF, 2 mM Tris-2-(N-morpholino)ethanesulfonic acid (MES), pH 7.2 (Sigma-Aldrich, St. Louis, MO, USA). The MF (500 µL) was layered onto a 25/38% discontinuous sucrose density gradient [700 µL 25% (*w*/*v*) and 700 µL 38% (*w*/*v*) sucrose, in 1 mM Tris-MES, pH 7.2 and 1 mM EDTA] prepared in 2 mL microcentrifuge tubes and centrifuged at 13,000× *g*, 4 °C for 1 h. The PM fraction was gently extracted from the 25/38% interface with a pipette and stored at −80 °C. Samples were subjected to acetone precipitation, and the concentration was quantified using the Amido Black assay method [50]. To validate the enrichment of the PM-associated fraction, Western blot analysis using anti-active MAP kinase (MAPK) antibody (Figure S1) and SDS-PAGE gel analysis were performed (data not shown).

### 2.5. Label-Free Liquid Chromatography-Mass Spectrometry Analysis

The sample clean-up and in-solution trypsin-digested Arabidopsis PM-associated protein samples were analysed at the Centre for Proteomic and Genomic Research (CPGR; Cape Town, South Africa).

#### 2.5.1. On-Bead Hydrophilic Interaction Liquid Chromatography (HILIC) Digest of In-Solution PM-Associated Protein Samples

PM-associated protein samples (1.25 mg/mL) were prepared by re-suspending acetone-precipitated proteins in a buffer containing 5% SDS in 50 mM ammonium bicarbonate, and the concentration was adjusted after quantification. MagResyn^®^ hydrophilic interaction liquid chromatography (HILIC) with iron oxide-containing magnetic polymer microparticles was used for sample clean-up and tryptic digestion pre-MS. This technique helps to remove possible contaminants in the samples and reagents before MS to reduce contaminant-induced data variability and improves reproducibility. The on-bead (HILIC) magnetic bead workflow was prepared after removing the shipping solution and aliquoting the supplier’s beads (Resyn Biosciences, Gauteng, South Africa) into a new tube. Beads were washed with 250 μL wash buffer containing 15% (*v*/*v*) acetonitrile (ACN; Sigma-Aldrich, St. Louis, MO, USA) and 100 mM ammonium acetate (Sigma-Aldrich, St. Louis, MO, USA), pH 4.5, for 1 min. This washing step was repeated once. The beads were then re-suspended in loading buffer containing 30% (*v*/*v*) ACN and 200 mM ammonium acetate, pH 4.5, to a concentration of 5 mg/mL. Proteins (50 μg) from each sample were transferred to a 96-well protein LoBind plate (Merck, Darmstadt, Germany) followed by reduction with tris (2-carboxyethyl) phosphine (TCEP; Sigma-Aldrich, St. Louis, MO, USA), which was added to a final concentration of 10 mM and incubated at 60 °C for 1 h.

Following the protein reduction, samples were cooled to room temperature and alkylated with methylmethane-ethiosulphonate (MMTS; Sigma-Aldrich, St. Louis, MO, USA), which was added to a final concentration of 10 mM and incubated at RT for 15 min. HILIC magnetic beads were added at an equal volume to that of the sample and a ratio of 5:1 (*v*/*v*) total protein. To enhance protein binding to the beads, the plate was incubated on a shaker at 900 rpm, RT for 30 min. The beads were then washed 4 times with 500 μL of 95% (*v*/*v*) ACN for 1 min. For on-bead digestion of the bound proteins, trypsin (Promega, Fitchburg, WI, USA), made up in 50 mM triethylammonium bicarbonate (TEAB), was added at a ratio of 1:12.5 (*v*/*v*) total protein and the plate was incubated at 37 °C on a shaker for 4 h. The microparticles were recovered on the magnetic separator followed by the collection of supernatant-containing peptides using a pipette. The peptide sample was dried and re-suspended in LC loading buffer (0.1% (*v*/*v*) formic acid (FA; Sigma-Aldrich, St. Louis, MO, USA) in 2.5% (*v*/*v*) CAN) and proceeded to LC-MS/MS analysis.

#### 2.5.2. LC-MS/MS Analysis

In-solution, trypsin-digested Arabidopsis PM-associated protein samples were analysed using the LC-MS system comprised of a Q-Exactive quadrupole-Orbitrap mass spectrometer (ThermoFischer Scientific, Waltham, MA, USA) coupled with a Dionex Ultimate 3000 nano-ultra performance liquid chromatograph (UPLC). Proteomic data was acquired using: Xcalibur v4.1.31.9, Chromeleon v6.8 (SR13), Orbitrap MS v2.9 (build 2926) and Thermo Foundations 3.1 (SP4). The mobile phase was solvent A (LC water (Burdick and Jackson, Michigan, USA) and 0.1% (*v*/*v*) FA) and solvent B (ACN and 0.1% (*v*/*v*) FA). Peptides in LC loading buffer (approximately 400 ng) were loaded on a C18 trap column (PepMap100, 300 μm × 5 mm × 5 μm). Samples were trapped onto the column at 2% solvent B and washed for 3 min before the valve was switched and peptides eluted onto the analytical column. Here, peptides were separated on a Waters nanoEase (Zenfit) M/Z Peptide CSH C18 column (75 μm × 25 cm × 1.7 μm) (Waters Corporation, Milford, MA, USA) using the multi-step gradient generated at 300 nL/min as follows: 2–5% solvent B for 5 min, 5–18% solvent B for 40 min, 18–30% solvent B for 10 min, 30–80% solvent B for 2 min, held at 80% solvent B for 10 min before returning to 2% solvent B for 5 min. A wash step was included at the end of the run (in order to ensure carryover did not occur) with the following gradient: 2–80% solvent B in 35 min, held at 80% solvent B for 5 min before returning to 2% solvent B and conditioning the column for 15 min. The mass spectrometer was operated in a positive ion mode with a capillary temperature of 320 °C. All data acquisition was performed using Proxeon stainless steel emitters (ThermoFischer, Waltham, MA, USA), and the applied electrospray voltage was 1.95 kV.

#### 2.5.3. Data Analysis

The database search was performed with the PMI-Byonic-com v2.6.46 Byonic^TM^ Software (Protein Metrics, Cupertino, CA, USA) using an Arabidopsis reference proteome sourced from UniProt Knowledgebase (UniProtKB) [51], whereby the spectra from peptide fragments resulting from MS/MS were matched. The following search parameters were used: collision induced dissociation (CID) low energy for fragmentation type, trypsin enzyme, cutting at the C-terminal of lysine and arginine residues, maximum number of missed cleavages of 2, fixed modification was carbidomethyl (M), and variable modifications were deamidated (NQ) and oxidation (methionine). The precursor mass tolerance was of 10 ppm and fragment mass tolerance was of 20 ppm, respectively. The protein false discovery rate (FDR) cut off was 1% and the best score range was between 0–1000, with anything larger than 300 considered significant. The software package generates unique peptides, representing the total number of peptide spectrum matches (PSMs) for the proteins. A score plot (representation of differential abundance of proteins) and the mass error loadings plot (depicting observed minus calculated *m/z*) were used to gauge possible variations, confidence and significance of identified proteins (Figure S2). A Byonic score (the primary indicator of PSM correctness) greater than 300 and log probability (log base 10 of the protein *p*-value) greater than 1 were used as thresholds to ascertain the significance of differentially expressed proteins [52]. These significant proteins were further searched against UniProtKB and the Arabidopsis information resource (TAIR) [53] to sieve for PM-associated proteins based on their subcellular location as well as GO molecular function and biological processes. Thus, significant proteins were grouped into functional categories. Furthermore, protein–protein interaction networks analysis of the significant proteins was performed using STRING database version 11.5 [54].

## 3. Results

### 3.1. LPS-Responsive PM-Associated Proteins in Arabidopsis WT, lbr2-2 and bak1-4

A label-free LC-MS/MS system previously shown to be effective in the study of MAMP-responsive plant proteomics [24,55] was used to identify PM-associated proteins in Arabidopsis WT, *lbr2-2* and *bak1-4* after treatment with LPS chemotypes from *Pst* and *Xcc*. Raw data generated from the LC-MS/MS were analysed with the Byonic^TM^ software and searched against UniprotKB alongside The Arabidopsis Information Resource (TAIR). Identified LPS-responsive PM-associated proteins with a Byonic score of >300 and log probability of >1 were compared to the control (MgCl_2_-treated Arabidopsis) proteins. The significant PM-associated proteins were functionally classified based on the GO molecular function and biological process (Table S1). These identified LPS-responsive PM-associated proteins were mainly involved in stress and defence responses, membrane transport and trafficking, perception and signalling, and metabolic processes. Those identified in cell structure, protein synthesis, transcription and others are not included in the discussion (but available on request).

### 3.2. Protein–Protein Interaction Network

The protein–protein interaction (PPI) networks associated with the LPS-responsive PM-associated proteins in Arabidopsis WT, *lbr2-2* and *bak1-4* were generated by the STRING database. In WT and mutants, proteins with similar functions formed PPI clusters (Figures S3–S8). In both LPS*_Pst_* and LPS*_Xcc_*-treated WT, three major clusters were observed, mainly involving proteins that participate in transport and trafficking, metabolic processes, and protein synthesis (Figures S3 and S4). For the LPS*_Pst_* and LPS*_Xcc_*-treated *lbr2-2*, there were four prominent clusters of PPIs showing proteins that are mainly involved in transport and trafficking, perception and signalling, metabolic process and protein synthesis (Figures S5 and S6). Lastly, while LPS*_Pst_*-treated *bak1-4* showed four major PPI clusters involved in transport and trafficking, perception and signalling, metabolic process and protein synthesis, the LPS*_Xcc_*-treated *bak1-4* showed several clusters containing the above stated functions and others (Figures S7 and S8). Overall, there were distinct PPI patterns observed in the LPS chemotypes-treated WT, *lbr2-2* and *bak1-4* plants, suggesting that knockout of LBR2 and BAK1 could have influenced the Arabidopsis responses to the two LPS chemotypes. For noting and clarity, the clustering differences are presented for visual significant variances only and not for the annotation of specific proteins (seen in small font).

### 3.3. Comparing Identified Proteins from Different Arabidopsis Lines

**Figure 1:**
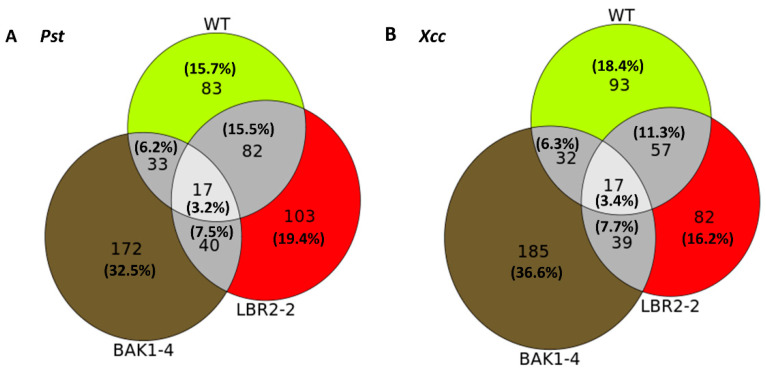
Venn diagram representations of how the groups of PM-associated proteins in the WT, *lbr2-2* and *bak1-4* plants relate to one another after treatment with LPS from *Pst* (**A**) and *Xcc* (**B**). The numbers in the sectors indicate the unique/distinct and overlapping/shared/common proteins.

Venn diagrams were created as schematic representations of the identified PM-associated proteins, distinct or shared in the WT, *lbr2-2* and *bak1-4* plants after LPS*_Pst_* and LPS*_Xcc_* treatments. Based on the differences and the similarities, proteins were categorised and compared, as shown in these visual representations (Figure 1).

For both LPS chemotypes, the highest number of distinct proteins were detected in the *bak1-4* mutant, followed by LPS*_Pst_*-treated *lbr2-2* and LPS*_Xcc_*-treated WT. In both LPS*_Pst_* and LPS*_Xcc_* treatments, similar numbers of proteins overlapped in the WT, *lbr2-2* and *bak1-4*. There were more overlapped proteins between WT and *lbr2-2* than any other pairs, for both LPS chemotype treatments. Although several LPS-responsive proteins were shared amongst the Arabidopsis WT and mutants (n = 17), there were more distinct proteins in the respective plants.

### 3.4. Comparison of the Distinct PM-Associated Proteins in Each Plant Line

**Figure 2:**
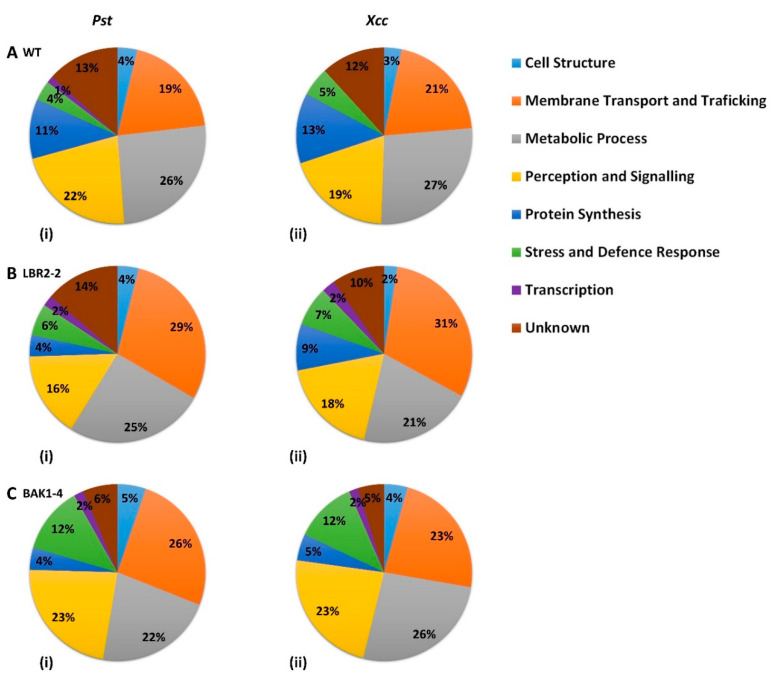
Pie chart comparison of functional categories of PM-associated proteins found to be distinctly associated with the response of the WT and two mutants to the LPS treatments. Proteins that were distinct to WT (**A**), *lbr2-2* (**B**) and *bak1-4* (**C**) after LPS from *Pst* (**i**) and *Xcc* (**ii**) were grouped based on their functional categories.

The distinct PM-associated proteins in the WT, *lbr2-2* and *bak1-4* plants as shown in the Venn diagrams (Figure 1) were selected and their functional categories compared (Figure 2A–C).

In both WT and mutants, three major classes of identified LPS-responsive distinct proteins were those involved in (i) metabolic processes, (ii) perception and signalling, and (iii) membrane transport and trafficking. Although proteins involved in protein synthesis were mostly observed in WT plants treated with both LPS chemotypes, a higher percentage of proteins involved in stress and defence responses were present in the mutants, particularly *bak1-4*. Furthermore, proteins distinct to WT treated with both LPS chemotypes showed a slightly higher percentage of proteins involved in metabolic processes compared to the mutants, whereas the percentage of proteins involved in membrane transport and trafficking were observed to be lower in the WT. The percentage of proteins involved in cell structure were comparable for all three lines treated with both LPS chemotypes, while *bak1-4* yielded the smallest percentage of proteins with unknown functions. Although the distinct proteins involved in transcription were less represented across the three lines, they were not detected in the LPS*_Xcc_*-treated WT. Finally, in both LPS chemotypes treatments, a higher percentage of distinct proteins involved in perception and signalling were observed in *bak1-4*, followed by WT and *lbr2-2*.

### 3.5. Total Peak Intensity Analysis of Selected PM-Associated Proteins

**Figure 3:**
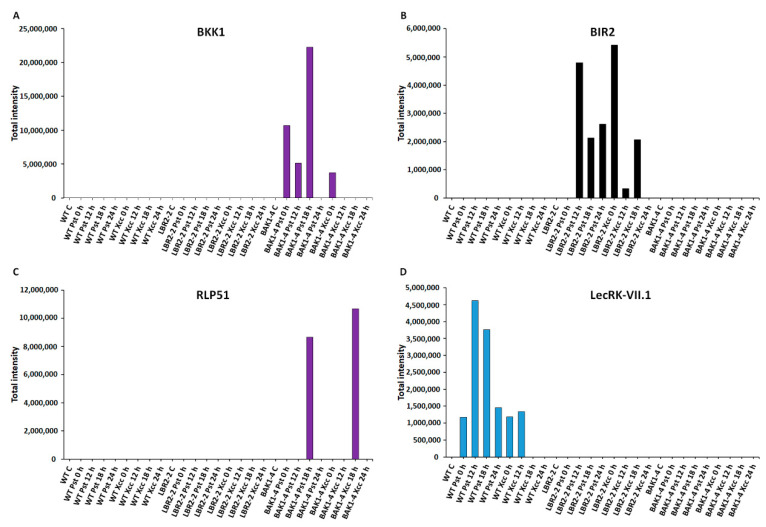
Comparative analyses of total peak intensities of PM-associated proteins involved in **perception and signalling**. Total peak intensity of selected significant PM-associated proteins for (**A**) BAK1-like 1 (BKK1), (**B**) BAK1-interacting receptor-like kinase 2 (BIR2), (**C**) Receptor-like protein 51 (RLP51) and (**D**) L-type lectin-domain containing receptor kinase VII.1 (LecRK-VII.1). Data from the WT are indicated in blue, the *lbr2-2* mutant in black and the *bak1-4* mutant in purple, with time periods of 0–24 h as indicated.

**Figure 4:**
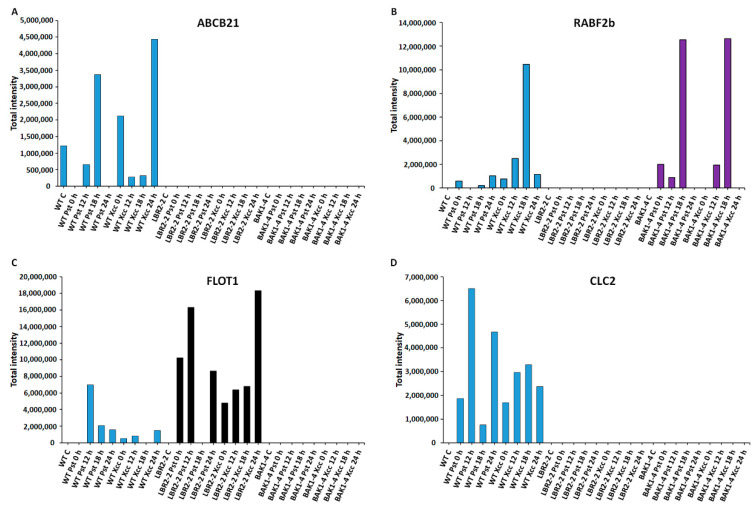
Comparative analyses of total peak intensities of PM-associated proteins involved in **membrane transport and trafficking**. Total peak intensity of selected significant PM-associated proteins for (**A**) ABC transporter B family member 21 (ABCB21), (**B**) Ras-related protein (RABF2b), (**C**) Flotillin-like protein 1 (FLOT1) and (**D**) Clathrin light chain 2 (CLC2). Data from the WT are indicated in blue, the *lbr2-2* mutant in black and the *bak1-4* mutant in purple, with time periods of 0–24 h as indicated.

**Figure 5:**
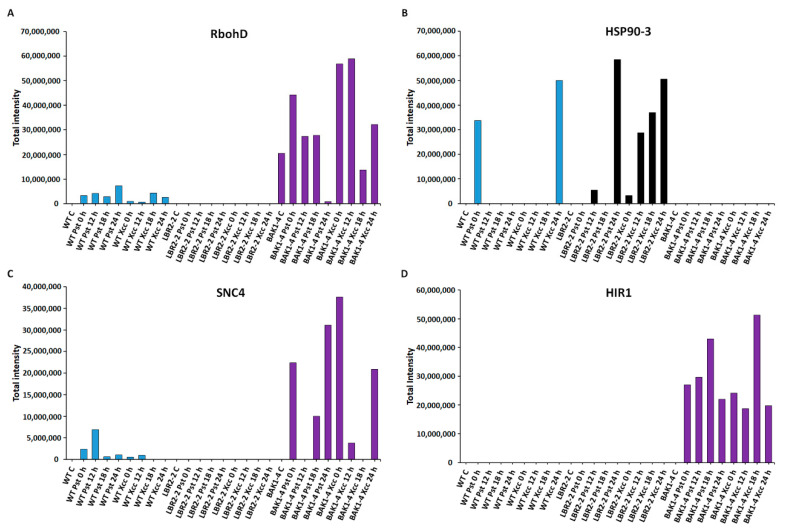
Comparative analyses of total peak intensities of PM-associated proteins involved in **stress and defence responses**. Total peak intensity of selected significant PM-associated proteins for (**A**) Respiratory burst oxidase homolog protein D (RbohD), (**B**) Heat shock protein 90-3 (HSP90-3), (**C**) Protein SUPPRESSOR OF NPR1-1 CONSTITUTIVE 4 (SNC4) and (**D**) Hypersensitive-induced response protein 1 (HIR1). Data from the WT are indicated in blue, the *lbr2-2* mutant in black and the *bak1-4* mutant in purple, with time periods of 0–24 h as indicated.

**Figure 6:**
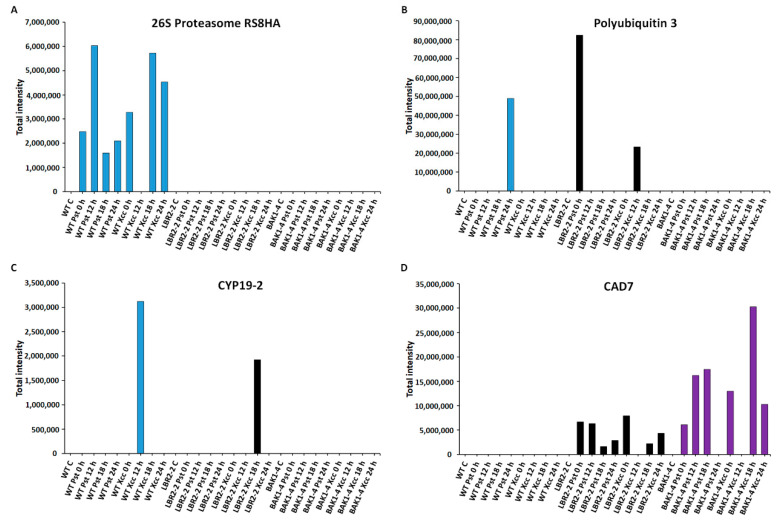
Comparative analyses of total peak intensities of PM-associated proteins involved in **metabolic processes**. Total peak intensity of selected significant PM-associated proteins for (**A**) 26S proteasome regulatory subunit 8 homolog A (26S Proteasome RS8HA), (**B**) Polyubiquitin 3, (**C**) Peptidyl-prolyl cis-trans isomerase (CYP19-2) and (**D**) Cinnamyl alcohol dehydrogenase 7 (CAD7). Data from the WT are indicated in blue, the *lbr2-2* mutant in black and the *bak1-4* mutant in purple, with time periods of 0–24 h as indicated.

As shown in Figure 3, Figure 4, Figure 5 and Figure 6, total peak intensities of selected LPS-responsive PM-associated proteins that are involved in perception and signalling (Figure 3), membrane transport and trafficking (Figure 4), stress and defence responses (Figure 5), and metabolic processes (Figure 6) were analysed. Notably, early and later LPS treatments represent (0–12 h) and (18–24 h) time points, respectively.

Figure 3A–D analysed proteins that are involved in **perception and signalling**, such as somatic embryogenesis receptor kinase 4 (SERK4)/BAK1-like 1 (BKK1), BAK1-interacting receptor-like kinase 2 (BIR2), receptor-like protein 51 (RLP51) and L-type lectin-domain containing receptor kinase VII.1 (LecRK-VII.1). The perception signalling function by receptors and/or co-receptors is key to the recognition of MAMPs during plant defence responses. In addition, PM-localised PRRs are either receptor-like proteins (RLPs) or receptor-like kinases (RLKs). As presented in Figure 3A, SERK4/BKK1 is a LRR-RLK protein, which is a BAK1 closest homolog that regulates MTI through association with PRRs [56,57]. Although BKK1 was not identified in the WT and *lbr2-2*, it showed an increased accumulation in *bak1-4* at early and 18 h LPS*_Pst_*-, and a lesser intensity at 0 h LPS*_Xcc_* treatments. Figure 3B shows an analysis of another LRR-RLK protein, BIR2, that regulates BAK1 during MTI [28]. BIR2 was not identified in WT and *bak1-4* but it increased at 12 h and later LPS*_Pst_*-, and at early and 18 h LPS*_Xcc_*-treated *lbr2-2*. As shown in Figure 3C, RLP51 is a LRR-RLP that represents RLP groups, some of which have been linked to the recognition of MAMPs during plant defences [36,58]. RLP51 was not identified in WT and *lbr2-2*. However, the case was different with the *bak1-4* mutant, which showed accumulation of RLP51 at 18 h in both LPS chemotypes treatments but was not detected in other time points. LecRK-VII.1 was selected due to the reported involvement of lectin domains in the recognition of ligands with carbohydrate moieties [59], which are also contained in the LPS molecular structure. L-type lectin-domain containing receptor kinase VII.1 (LecRK-VII.1) accumulated only in the LPS*_Pst_*-treated WT but was not identified in *lbr2-2* and *bak1-4* (Figure 3D).

Four LPS-responsive proteins involved in **membrane transport and trafficking** were selected, and the total intensities analysed (Figure 4A–D). ABC transporter B family member 21 (ABCB21) was selected as the representative of ABC transporters family proteins involved in the transport of lipids, secondary metabolites and hormones [60]. As shown in Figure 4A, ABCB21 was identified only in the WT with increased accumulation at 18 h LPS*_Pst_* treatment and at 0 h and 24 h LPS*_Xcc_* treatments. This protein was not identified in *lbr2-2* and *bak1-4*. Ras-related protein (RABF2b) is part of the RAB proteins with established protein trafficking functions [61]. As presented in Figure 4B, RABF2b increased at 24 h LPS*_Pst_*-treated and at 12 and 18 h LPS*_Xcc_*-treated WT. In addition, RABF2b was not identified in the *lbr2-2* but it showed increased accumulation in *bak1-4,* especially at both 18 h LPS*_Pst_* and LPS*_Xcc_* treatments. Flotillin-like protein 1 (FLOT1) and clathrin light chain 2 (CLC2) have been shown to play key roles in protein internalisation during endocytosis [62,63]. FLOT showed increased accumulation at 12 h LPS*_Pst_* treatment but decreased at later time points in the WT (Figure 4C). Overall, there was less accumulation of FLOT1 in the LPS*_Xcc_*-treated WT compared to the LPS*_Pst_* treatment. For *lbr2-2*, FLOT increased in LPS*_Pst_* treatment but was not detected at 18 h and showed reduction at 24 h. However, when treated with LPS*_Xcc_* the protein increased up until 24 h. Notably, FLOT was not identified in *bak1-4*. On the other hand, CLC2 was only identified in the WT (Figure 4D). It showed increased accumulation in both early and later time points in both LPS*_Pst_* and LPS*_Xcc_* treatments.

Figure 5A–D shows an analysis of the proteins involved in **stress and defence responses**. Respiratory burst oxidase homolog protein D (RbohD) is instrumental in the reactive oxygen species (ROS) burst production, a part of plant’s early defence responses [40]. As presented in Figure 5A, RbohD accumulated in both LPS*_Pst_* and LPS*_Xcc_*-treated WT especially at 24 h and 18 h, respectively. Although RbohD was not identified in *lbr2-2*, it showed increased accumulation at early and 18 h LPS*_Pst_*-, and at early and 24 h LPS*_Xcc_* treatments in *bak1-4*. Heat shock protein 90-3 (HSP90-3) is part of HSPs with activities including protein folding during plant stress [64]. As shown in Figure 5B, HSP90-3 increased at 0 h LPS*_Pst_* and 24 h LPS*_Xcc_*-treated WT. HSP90-3 was not identified in *bak1-4* but it showed higher accumulation in *lbr2-2* at 24 h in both LPS*_Pst_* and LPS*_Xcc_* treatments. Protein SUPPRESSOR OF NPR1-1 CONSTITUTIVE 4 (SNC4) is part of R proteins with defence roles against effector-mediated defence responses against bacteria [65]. SNC4 showed increased accumulation at 12 h LPS*_Pst_*-treated WT but was not identified in the *lbr2-2* (Figure 5C). Although SNC4 accumulated at 0 h and later time points in the LPS*_Pst_*-treated *bak1-4*, it was accumulated at early and 24 h LPS*_Xcc_* treatment. Hypersensitive-induced response protein 1 (HIR1) belongs to the group of proteins linked to the hypersensitive response that characterises ETI [66,67]. HIR1 was not identified in the WT and *lbr2-2* plants but showed increased accumulation at both early and late time points in the LPS*_Pst_* and LPS*_Xcc_* treatments of *bak1-4*, especially at the 18 h (Figure 5D).

Finally, the total intensities of the PM-associated proteins involved in **metabolic processes** such as 26S proteasome regulatory subunit 8 homolog A (26S proteasome RS8HA), polyubiquitin 3, peptidyl-prolyl cis-trans isomerase (CYP19-2) and cinnamyl alcohol dehydrogenase 7 (CAD7) were analysed as shown (Figure 6A–D). The 26S proteasome regulatory subunit 8 homolog A and polyubiquitin 3 are part of the 26S proteasome-ubiquitin (UPS) system and play vital roles in the regulation of protein degradation and turnover [68]. As shown in Figure 6A, 26S proteasome regulatory subunit 8 homolog A was not identified in *lbr2-2* and *bak1-4*. This protein showed increased accumulation at both early and later LPS*_Pst_*- and LPS*_Xcc_*-treated WT especially at 12 h and 18 h, respectively. On the other hand, polyubiquitin 3 was not identified in the *bak1-4*, but it was accumulated at 24 h LPS*_Pst_*-treated WT and at 0 h LPS*_Pst_* and 12 h LPS*_Xcc_*-treated *lbr2-2* (Figure 6B). Peptidyl-prolyl cis-trans isomerase (CYP19-2) is part of cyclophilins (CYPs), which play a regulatory role in protein degradation and stress responsiveness [69]. CYP19-2 was not identified in the *bak1-4* but was accumulated at 12 h LPS*_Pst_*-treated WT and at 18 h LPS*_Xcc_*-treated *lbr2-2* (Figure 6C). Cinnamyl alcohol dehydrogenase 7 (CAD7) is a member of alcohol dehydrogenases that catalyse the biosynthesis of monolignol-derived secondary metabolites involved in defence against pathogens [70,71]. As presented in Figure 6D, CAD7 was not identified in the WT but in *lbr2-2* it displayed more accumulation at early LPS*_Pst_*-, and decreased accumulation at a later treatment. Similarly, this protein showed more accumulation at 0 h LPS*_Pst_* when compared to the later time points after LPS*_Xcc_* treatments. There was an increased accumulation of CAD7 at early and 18 h LPS*_Pst_*-treated *bak1-4* and at 0 h and later time points in the LPS*_Xcc_* treatment, especially at the 18 h mark.

## 4. Discussion

The PM mediates communication between the external and internal milieus with the aid of proteins embedded in the membrane phospholipid bilayer. Membrane rafts within the PM are potential hubs for specific receptor and co-receptor complex assembly and signalling output [72,73]. To date, the PRRs and/or co-receptor complexes for LPS linked to its associated defence signalling is debatable. Identification of LPS-responsive proteins in the PM and those loosely associated/bound thereto will give insight into the molecular mechanism associated with LPS perception and signalling. In this study, a label-free LC-MS/MS system aided in the identification of LPS-responsive PM-associated proteins in Arabidopsis WT, *lbr2-2* and *bak1-4* plants, showing nuanced differences between the three lines. These proteins are discussed in accordance with four functional categories, namely (i) perception and signalling, (ii) membrane transport and trafficking, (iii) defence and stress responses, and (iv) metabolic processes (Table S1; summarised in Table 1).

### 4.1. PM-Associated Proteins Related to Perception and Signalling

PRRs (either RLKs or RLPs), use different conserved ectodomains, including leucine-rich repeat (LRR), lysine motif (LysM) or lectin domains (Lec), to perceive MAMPs prior to activation of defence signalling [3]. The effective concentration of RLKs on the PM can be highly dynamic, up-regulated by MAMPs, trapped in membrane rafts and in complexes with functionally related proteins, as well as subject to PM clearance by endocytosis and recycling [74]. Several PM-localised LPS-responsive LRR-RLKs and LRR-RLPs were identified in this study (Table 1 and Table S1). Somatic embryogenesis receptor kinases (SERKs) are LRR-RLK proteins involved in plant signalling mainly through ligand-induced dimerisation with PRRs [75]. Here, SERK2 was identified only in 12 h LPS*_Xcc_*-treated WT (Table 1 and Table S1). SERK2, a close homolog of SERK1 has been shown to be highly expressed during plant development [76,77]. On the other hand, SERK4/BAK1-like1 (BKK1) was identified in *bak1-4* at early and 18 h LPS*_Pst_* treatment. BAK1/SERK3 and its closest homolog and BKK1 have dual physiological roles of positively regulating the brassinosteroid (BR)-dependent growth pathway and negatively regulating the BR-independent cell death in a salicylic acid (SA)-dependent manner [56,78]. Figure 3A shows BKK1 accumulation mainly in the LPS*_Pst_*-treated *bak1-4* compared to 0 h only observed in the LPS*_Xcc_* treatment. Notably, while BKK1 was not identified in the WT and *lbr2-2*, its accumulation in the *bak1-4* mutant suggests functional redundancy to compensate for the loss of BAK1 (closest homolog of BKK1).

Constitutive defence responses can consume metabolic energy and cellular resources and can be detrimental to the plant, as such the defence signalling needs to be negatively regulated. The Arabidopsis LRR-RLKs BAK1-interacting receptor-like kinase 1 (BIR1), BIR2 and BIR3 function as negative regulators of MAMP-triggered immune signalling [29,79,80,81]. In this study, BIR1 was identified at later time points in *lbr2-2* treated with both LPS chemotypes, while BIR2 was identified early as well as later in the case of the LPS*_Pst_* treatment. In addition, BIR3 was identified in both *lbr2-2* and *bak1-4* mutants treated with both LPS chemotypes, at early and later time points (Table 1). Thus, no BIR protein was identified in WT, while all BIR (BIR1, BIR2 and BIR3) were identified in *lbr2-2*. Furthermore, as presented in Figure 3B, while BIR2 was not identified in WT and *bak1-4*, it was accumulated in LPS*_Pst_*-treated *lbr2-2*. Upon MAMP perception by PRRs, there is a quick dissociation of BIR1 from the BAK1–BIR1 complex, which allows BAK1 to form an active complex with SUPPRESSOR OF BIR1-1 (SOBIR1), leading to downstream cell death and defence signalling [80]. Here, a LRR-RLK SOBIR1 was identified at 12 h in the LPS*_Pst_*-treated WT. SOBIR1, an adaptor to RLPs regulates cell death and innate immunity in Arabidopsis [80,81,82]. In a BIR1 loss-of-function study, SOBIR1 was reported to interact with BAK1 and promote cell death in Arabidopsis [80].

The LRR-RLP RLP23 forms a complex with SOBIR1 in a ligand-independent manner and recruits BAK1 into the complex upon treatment with nlp peptides (conserved amino acid fragments found in numerous bacterial, fungal and oomycete necrosis and ethylene-inducing peptide 1-like proteins (NLPs)), and activates immune responses in Arabidopsis [83,84,85]. Furthermore, the expression of RLP23 in potato (*Solanum tuberosum*) confers nlp20 recognition that leads to improved immunity against oomycetal *Phytophthora infestans* and fungal *Slerotinia sclerotiorum* pathogens [84]. In this study, a putative disease resistance protein (RLP23) was identified at early and later time points in LPS*_Pst_*-treated *lbr2-2*, as well as at a later time point when treated with LPS*_Xcc_*. Furthermore, RLP23 was identified at early time points in both LPS*_Pst_*- and LPS*_Xcc_*-treated *bak1-4* (Table 1 and Table S1). Another RLP, RLP51/suppressor of npr1-1, constitutive 2 (SNC2), identified at 18 h in *bak1-4* treated with both LPS chemotypes showed increased accumulation at the said time point but was not identified in WT and *lbr2-2* (Figure 3C). The lack of increased accumulation of RLP51 in the Arabidopsis *lbr2-2* mutant could be as a consequence of the knockout of LBR2, which possibly could attribute to the attenuation of LPS recognition and signalling. Furthermore, the accumulation of RLP51 observed at 18 h in both LPS*_Pst_*- and LPS *_Xcc_*-treated *bak1-4* suggests a possible involvement of BAK1’s closest homologue as stated above in the LPS signalling. A study has demonstrated the involvement of Arabidopsis RLP51 in basal resistance against *Pst* [86]. These identified LRR-RLKs and LRR-RLPs suggest a possible involvement of receptors or co-receptors with LRR external ectodomains in LPS perception and signalling in Arabidopsis.

Lysin motif (LysM)-containing cell surface receptors which are either (LysM)-containing receptor-like kinases (LYKs) or (LysM)-containing receptor-like proteins (LYKPs) are involved in the perception of specific microbe-derived oligosaccharide elicitors. These include chitin and peptidoglycan (PGN) via the N-acetylglucosamine (GlcNAc) carbohydrate moiety and induce immune responses in plants [87,88,89,90,91]. In this study, a LysM-RLK chitin elicitor receptor kinase 1 (CERK1) was identified at 24 h in LPS*_Xcc_*-treated *lbr2-2* and at early LPS*_Pst_* treatments as well as at early and later LPS*_Xcc_*-treated *bak1-4* (Table 1 and Table S1). CERK1 is a well-characterised immune receptor for the fungal chitin elicitor (chitooligosacharides) as well as bacterial PGNs [38]. OsCERK1 forms a complex with LYP CEBiP that lacks a cytoplasmic kinase activity to trigger chitin-dependent defence signalling in rice [92]. Furthermore, OsCERK1 has been shown to participate in LPS perception and associated immune response in rice [93]. Here, LysM domain receptor-like kinase 3 (LYK3) was identified at 24 h in LPS*_Xcc_*-treated *lbr2-2* and at early time points in LPS*_Pst_*-treated *bak1-4*. Another LYK, LysM domain receptor-like kinase 4 (LYK4) was identified at 18 h in LPS*_Pst_*-treated *bak1-4*. Although LYK4 is involved in chitin perception and defence signalling in Arabidopsis [94], LYK3 acts as a *Sinorhozobium meliloti* root nodule symbiotic (Nod factor signals) receptor in *Medicago truncatula* [95]. Studies have shown that Arabidopsis LYK3 is required for the Nod factor-induced suppression of MTI and resistance against *Botrytis cinerea* and *Pectobacterium carotovorum* [96,97]. Considering the LPS-responsive PM-localised LysM-RLK proteins identified in this study, especially those that participate in chitin recognition, it may be interesting to further investigate the possible role of these classes of proteins in LPS recognition and defence signalling.

The highly variable nature of lectin domains allows the lectin (Lec) receptor kinases (LecRKs) to recognise a wide range of saccharide ligands and participate in plant–pathogen interactions [59,98]. Sanabria et al. [99] reported LPS-induced expression of the S-domain G-type lectin receptor kinase (*Nt-Sd-RLK*) gene in *Nicotiana tobacum*. In this study, we also identified G-type LecRKs (Table S1). G-type lectin S-RLK At1g61440 and G-type lectin S-RLK At4g03230 were identified at 18 h and 12 h in LPS*_Xcc_*-treated *bak1-4*. Moreover, G-type lectin S-RLK At2g19130 was identified in *lbr2-2* at early time points when treated with LPS*_Pst_.* On the other hand, four L-type lectin-domain containing RLKs were identified in this study (Table S1). For example, L-type lectin-domain containing RLK IV.4 was identified at both early and later time points in *bak1-4* when treated with both LPS chemotypes. Although L-type lectin-domain containing RLK S.1 was identified in all three lines (at 24 h LPS*_Xcc_*-treated WT, and 12 h LPS*_Pst_*-treated *lbr2-2* and *bak1-4*), L-type lectin-domain containing RLK VII.1 (LecRK-VII.1) was identified at 18 h in LPS*_Pst_*-treated WT only (Table 1 and Table S1). LecRK-VII.1 showed increased accumulation in WT but not in mutants, as shown in Figure 3D. In fact, LecRK-VII.1 was not detected in LPS-treated *bak1-4* and *lbr2-2*, further suggesting a possible role of LBR2 and BAK1 in LPS perception and signalling. Using enrichment and label-free LC-MS/MS strategies, candidate LPS-interacting LecRKs were also identified in our group [24,30]. Since lectins are carbohydrate-binding proteins, it is tempting to speculate the involvement of LecRKs in the perception and signalling of LPS given its lipoglycan nature.

Cysteine (Cys)-rich RLKs (CRKs) are membrane-localised RLKs with extracellular Cys-rich repeats (CRR) that contain a novel C-X8-C-X2-C motif which participates in defence signalling [100,101]. Upon flg22 perception, Arabidopsis CRK28 self-associates and also associates with closely related CRK29 as well as the FLS2–BAK1 complex to enhance plant immune response [102]. In another study, CRK36 formed a complex with FLS2 and the Botrytis-induced kinase 1 (BIK1) upon flg22 recognition and regulates defence responses [100]. In this study, we identified thirteen CRKs that might be playing roles in the perception and signalling in response to LPS as a MAMP (Table 1 and Table S1). For instance, CRK RLK CRK17 was identified in all three lines. For WT, it was identified at 18 h in LPS*_Pst_*-treatment, and in both early and later time points when treated with LPS*_Xcc_*. In addition, while CRK17 was identified at early and later time points in both LPS*_Pst_*- and LPS*_Xcc_*-treated *lbr2-2*, it was identified at early treatments in *bak1-4*. CRK2 was identified at 12 h in LPS*_Pst_*-treated WT and *lbr2-2*, and at later time points in these plants after LPS*_Xcc_* treatment. CRK6 and CRK8 were both identified at 18 h and 12 h in LPS*_Pst_*- and LPS*_Xcc_*-treated *bak1-4*. Although CRK19 and CRK4 were both identified only in LPS*_Pst_*-treated *lbr2-2*, CRK21 was identified only at the early time point in *bak1-4*. Moreover, putative CRK12 was identified at only 24 h in LPS*_Pst_*-treated WT, and putative CRK31 was identified in response to both LPS chemotype treatments. Finally, putative CRK30 was identified in both LPS chemotypes-treated WT as well as at later time points in LPS*_Xcc_*-treated *lbr2-2*. Yeh et al. [103] demonstrated that Arabidopsis CRK4, CRK6, and CRK36 associated with FLS2 and enhanced resistance to bacterial *Pst* DC3000 in an flg22-dependent manner. CRK2 has been reported to activate RbohD and regulate MTI-triggered ROS burst [104]. Using transcriptomics, Iizasa et al. [105] identified LPS-induced LBR2-dependent genes that code for CRK5 and CRK38 proteins in Arabidopsis. Considering the large number of CRKs identified in this present study and their reported roles, their involvement in LPS defence signalling in Arabidopsis could be speculated on.

CDPKs play a key role in the regulation of plant innate immunity [106]. For instance, a mycelial wall-released elicitor from fungal pathogen *Fusarium oxysporum* f. sp. *matthiolae* induced CDPK1-dependent SA accumulation and enhanced disease resistance against pathogens [107]. Nine CDPKs were identified in this study (Table 1 and Table S1). CDPK29 and CDPK3 were identified at early time points in the LPS*_Pst_-* and LPS*_Xcc_*-treated *bak1-4* and *lbr2-2*, respectively. CDPK32 and its closest homologue CDPK33 were only identified in WT, with CDPK32 identified at early and later LPS*_Pst_* time points and later LPS*_Xcc_* time points, while CDPK33 was identified at 12 h in LPS*_Xcc_* treatment. Similarly, CDPK7 and its closes homologue CDPK8 were identified at 12 h in LPS*_Pst_*-treated WT. These similar patterns of identifying close homologues, such as CDPK32 and CDPK33 as well as CDPK7 and CDPK8 only in WT, suggests similar activities and possible redundancy amongst CDPKs function during MTI. CDPK5 identified at 12 h in LPS*_Pst_*-treated WT, as well as in both LPS chemotypes-treated *lbr2-2* (at 12 h and 24 h in LPS*_Pst_* treatments and at 24 h in LPS*_Xcc_* treatments) and *bak1-4* (at early and later time points after LPS*_Xcc_* treatments) has been shown to directly phosphorylate RbohD and enhance SA-mediated ROS burst, defence gene expression and resistance to *Pst* upon PAMP stimulation [108]. In our group, several LPS-responsive CDPKs have previously been identified in Arabidopsis [24,30,31]. Since LPS-induced CDPKs have been consistently identified from Arabidopsis using different proteomics studies in our group, these proteins most likely participate in LPS defence signalling. Nevertheless, further studies will be required to confirm CDPKs involvement in LPS early signalling as it has been documented in flg22-mediated MTI [109].

Plants also encode RLCKs that are typically recruited by PRRs to transduce perceived signals to downstream signalling components [110,111]. The Arabidopsis RLCKs seemingly are involved in a variety of signalling events related to stress responses [112] broadly required for the signalling of multiple PRRs [113] and may inhibit or enhance innate immune responses [22,114]. Several LPS-induced PBLs were identified in this study (Table 1 and Table S1). For example, probable serine/threonine-protein kinase (S/T-PK) PBL1 was identified at 18 h in LPS*_Xcc_*-treated *bak1-4*, whereas PBL7, PBL11 and PBL24 were identified at 18 h, 24 h and 12 h in LPS*_Xcc_*-treated *lbr2-2*, respectively. PBL22 was identified at 12 h and 24 h in LPS*_Pst_*- and LPS*_Xcc_*-treated *lbr2-2*, respectively, the S/T-PK PBL27 was identified at 18 h in LPS*_Pst_*- and LPS*_Xcc_*-treated WT (Table 1 and Table S1). A study has shown that PBL1 and BIK1, all from the RLCK VII subfamily, associate with FLS2 upon flg22 perception and regulate immunity [111]. The S/T-PK PBL27 is reported to interact with CERK1 in the absence of chitin ligand but upon chitin perception, it is phosphorylated by CERK1 and itself phosphorylates MAP kinase MAPKKK5 at the PM and, by so doing, connects CERK1-LYK5 to MAPK signalling [115]. Another Arabidopsis RLCKs PBL2 identified in this study and RPM1-induced protein kinase (RIPK) are involved in the XopAC type III effector-induced defence against black rot-causing bacterial *Xcc* [116]. S/T-PK RIPK was identified in the WT treated with both LPS chemotypes (at early LPS*_Pst_*, and both early and later LPS*_Xcc_* treatments) as well as in the later time points in the LPS*_Pst_*-treated *lbr2-2*.

Brassinosteroid (BR) signalling kinases (BSKs) are RLCKs that activate BR signalling downstream of BRI1 [117]. BSKs have also been implicated in the regulation of plant defence responses [118,119]. Five BSKs were identified in this study (Table 1 and Table S1). For example, S/T-PK BSK3 was only identified at later LPS*_Xcc_*-treated WT while BSK6 was identified at 12 h in LPS*_Pst_*-treated *bak1-4*. Moreover, BSK2 was identified at 24 h in LPS*_Pst_*-treated WT and 18 h in LPS*_Xcc_*-treated *bak1-4*. BSK5 was identified in WT and *bak1-4* at 12 h in LPS*_Pst_* and LPS*_Xcc_* treatments, respectively. BSK8 identified at 12 h in LPS*_Xcc_*-treated WT and its closely related BSK7 have been shown to interact with FLS2 and regulate flg22-induced MTI responses in Arabidopsis [119]. Xu et al. [120] showed that BSK3 interacted in vivo with several RLKs that participate in plant immunity. On the other hand, BSK5 is reported to associate with PRRs and trigger defence responses upon flg22, elf18 and pep1 MAMPs/DAMPs perception in Arabidopsis [118]. RLCKs are targeted by bacterial effector molecules and regulate plant immune responses [111]. Taken together the RLCKs identified from this study with well-established roles in both MTI and ETI, it suggests there is a possibility that these proteins may play key roles in LPS defence signalling in Arabidopsis.

### 4.2. PM-Associated Proteins Related to Membrane Transport and Trafficking

Since plant PMs act as the frontline for cellular import and export of chemicals, transport and trafficking are vital phenomena during plant defence responses against pathogens [121]. Pathogens on the other hand have developed the means to manipulate membrane transport and vesicle trafficking to inhibit specific plant responses [122,123]. In this study, several proteins that participate in membrane transport and trafficking were identified, including ABC transporters, aquaporin, exocyst complex subunits, ras-related proteins, flotillin-like proteins, syntaxin, patellin and others (Table 1 and Table S1). These proteins have been reported to regulate different physiological activities in plants, including vesicle trafficking, endocytosis and transport of secreted defence-related phytohormones and secondary metabolites to target location [60,63,124,125,126].

Eleven ABC transporters were identified in this study with 7 B family members, 1 C family member, 1 F family member and 2 G family members (Table 1 and Table S1). For instance, ABC transporter B family member 21 and ABC transporter G family member 32 were identified only in Arabidopsis WT. The former was identified at 18 h in LPS*_Pst_*-, and at 12 h and 18 h in LPS*_Xcc_* treatments, while the latter was identified at 12 h in LPS*_Pst_*-, and at 12 h and 18 h in LPS*_Xcc_* treatments, respectively. ABC transporter B family member 17, ABC transporter B family member 29 chloroplastic and ABC transporter G family member 12 were identified only in *lbr2-2*. Whereas ABC transporter B family member 17 and ABC transporter B family member 29 chloroplastic were identified at later LPS*_Pst_*-treated time points in *lbr2-2*, ABC transporter G family member 12 was identified at 12 h in LPS*_Pst_* treatment. Whereas ABC transporter B family member 20 was identified at 18 h in LPS*_Pst_*-treated *bak1-4*, ABC transporter C family member 4 was identified at 18 h in both LPS chemotypes-treated *bak1-4*. As shown in Figure 4A, while ABC transporter B family member 21 was not identified in *lbr2-2* and *bak1-4*, it showed increased accumulation in both LPS chemotypes-treated WT. ABC transporter ABCG36/PEN3 has been linked to flg22-mediated defence response against microbial pathogens in Arabidopsis [124]. ABC transporter B family 4 was identified in both LPS chemotypes-treated WT, and LPS*_Xcc_*-treated *bak1-4* was reported as a LPS-responsive transporter by Vilakazi et al. [31]. ABCG34 has been shown to mediate the secretion of camalexin, a major phytoalexin in Arabidopsis and prevents infection against the necrotrophic pathogen *Alternaria brassicicola* [127].

Aquaporins of the PM intrinsic protein (PIP) family, typically known as water transporting channels, are also involved in the regulation of plant immunity against infections [122]. PIPs are involved in substrate transportation between cells interior and exterior as against other aquaporins families that transport between organelles [122,128,129]. Some aquaporins were identified in this study (Table 1 and Table S1). Although aquaporin PIP2-3 was identified in WT and treated with both LPS chemotypes, LPS*_Xcc_*-treated *lbr2-2* and LPS*_Pst_*-treated *bak1-4*, aquaporin PIP1-3 was only identified in WT and *lbr2-2* for both chemotypes (Table 1). Arabidopsis probable aquaporin PIP1-4 identified at early and later time points in both LPS chemotypes-treated *lbr2-2* and *bak1-4* has been shown to play a key role in the transport of apoplastic hydrogen peroxide (H_2_O_2_) across the PM and regulates MTI and systemic acquired resistance (SAR) [129,130]. PIP1-3 identified in both LPS-treated WT (at 24 h LPS*_Pst_* and 12 h and 18 h LPS*_Xcc_* treatment) and *lbr2-2* (at early and later time points LPS*_Pst_* treatments and at later time points in LPS*_Xcc_* treatments) has been shown to regulate defence responses in rice by interacting with pathogenic bacterial translocator the harpin protein, Hpa1, in the cytosolic import of the transcription activator-like effector PthXo1 [131].

The Rab family of small GTPases, which represent the largest branch of the Ras superfamily, mediate intracellular membrane trafficking via several means, such as transport and tethering of membrane-bound vesicles to the membrane, and as such regulates plant responses to biotic stressors [61,132]. These Rab GTPases play defence roles by participating in the secretion of defence-related proteins such as pathogenesis-related (PR) proteins to the PM or other endomembrane compartments as well as in PRR degradation or recycling [63,121]. Arabidopsis RABA1 GTPases have been reported to play a key role in transport between the trans-Golgi network and PM during tolerance against salinity stress [133]. Arabidopsis RAB proteins consist of eight groups: RABA, RABB, RABC, RABD, RABE, RABF, RABG, and RABH, with RABA consisting as many as 26 members out of the total of 57 RABs [133,134]. Twenty-seven LPS-responsive Ras-related proteins were identified in this study which comprises 13 RABA, 1 RABB, 1 RABC, 4 RABD, 3 RABE, 2 RABF, 3 RABG (Table 1 and Table S1). Here, 7 RAB protein groups (groups A–G) were identified except for RABH. Notably, most RAB proteins were identified in and shared between WT and *lbr2-2* (Table 1). Members of RABA, RABD and RABE groups were distributed amongst all lines, whereas RABB (Ras-related protein RABB1c) was identified only in both LPS chemotypes-treated *lbr2-2*, and RABC (Ras-related protein RABC1) was identified in both LPS chemotypes-treated WT and *lbr2-2*. Finally, while RABF proteins were distributed amongst WT and *bak1-4* treated with both LPS chemotypes, RABG proteins were distributed in WT and *lbr2-2*. In addition, RABF2b was not identified in *lbr2-2* but showed increased accumulation in the later time points in the LPS*_Xcc_*-treated WT as well as the LPS*_Pst_*- and LPS*_Xcc_*-treated *bak1-4* (Figure 4B). Overall wide distribution pattern of RAB proteins observed in WT and mutants suggests the common and distinct roles of these protein subclasses in LPS chemotypes perception and signalling.

SNARE (soluble N-ethylmaleimide-sensitive fusion protein attachment protein receptors) can form complexes with synthaxin proteins and regulate secretory trafficking to the PM and concomitant immune responses against pathogens [135]. Here, syntaxin-122 was identified at early and later time points in both LPS chemotypes-treated *bak1-4*. Several LPS-responsive exocyst complex subunits were identified in this study (Table 1 and Table S1). The exocyst subunits are proposed to exist as subcomplexes that mediate early steps of exocytosis through mediating vesicle tethering to PM membrane before SNARE-mediated fusion and play a vital role in response to biotic stresses [126,136]. Selected Exo70 paralogs including Exo70A1 identified at 18 h in LPS*_Xcc_*-treated WT and 12 h in LPS*_Pst_*-treated *lbr2-2* are involved in plant–biotic interactions [126]. In addition, Arabidopsis Exo70B1 identified at 12 h in LPS*_Pst_*-treated *bak1-4* is involved in the defence response against different pathogens [137]. Upon flg22 recognition, Exo70B1 and Exo70B2 regulate the trafficking of FLS2 to the PM [138], and the former is targeted by the U-box-type ubiquitin ligase 22 (PUB22) [136]. Exocyst complex subunits Exo70B2 and Exo70H1 are involved in the defence response against *P. syringae* pv. *maculicola* pathogen attack [139]. Flotillin is one of the plasma membrane lipid rafts markers with roles in endocytic trafficking pathways after internalisation from the PM [62,140]. In this study, flotillin-like protein 1 was identified in WT (at early and later LPS*_Pst_* treatments and at 24 h in LPS*_Xcc_* treatment) and *lbr2-2* (at early and 24 h in LPS*_Pst_* and at later LPS*_Xcc_* treatments) (Table 1 and Table S1). As shown in Figure 4C, flotillin-like protein 1 was not detected in *bak1-4* but was accumulated in WT and *lbr2-2* in both LPS*_Pst_-* and LPS*_Xcc_* treatments. Moreover, flotillin-like protein 2 was only identified at early and 24 h in LPS*_Pst_*-treated WT and at 24 h in LPS*_Xcc_* treatments. Flotillin 1 is a membrane raft-associated protein which has been shown to be involved in a clathrin-independent endocytic pathway in Arabidopsis [141]. Clathrin light and heavy chains are key components of the clathrin-coated vesicles that facilitate bound ligand internalisation. Clathrin heavy chain 1 was identified in both LPS chemotypes-treated WT (at 12 h in LPS*_Pst_* treatment and later LPS*_Xcc_* treatments) and *bak1-4* (at early and later LPS*_Pst_*- and LPS*_Xcc_* treatments) while clathrin heavy chain 2 was identified at 24 h in LPS*_Xcc_*-treated *bak1-4* (Table 1 and Table S1). Moreover, clathrin light chain 1 was identified in both LPS chemotypes-treated *lbr2-2* (at early and later LPS*_Pst_-* and LPS*_Xcc_* treatments) while clathrin light chain 2 was identified only at 12 h in LPS*_Pst_-* and 24 h in LPS*_Xcc_*-treated WT. Figure 4D, further shows that clathrin light chain 2 was accumulated in WT but not detected in the mutants. It has been reported that the internalisation of immune-activated PRRs, such as FLS2, EFR and PEPR1/2, into the endosomes requires clathrin proteins [142]. SA regulates endocytosis by interfering with clathrin-mediated protein trafficking but did not affect flg22-induced endocytosis of the FLS2 receptor during pathogen responses in Arabidopsis [143]. Mgcina et al. [144] proposed that LPS binding might involve ligand-induced receptor endocytosis and recycling in Arabidopsis. Endocytosis of PM-localised PRR for recycling or degradation involving transport and trafficking proteins is part of the regulation of ligand-induced defence signalling [63,121]. These reported findings and results from this study suggest that LPS chemotypes may trigger the production of proteins that participate in transport and trafficking in the Arabidopsis.

### 4.3. PM-Associated Proteins Related to Stress and Defence Response

In this study, the respiratory burst oxidase homolog protein D (RbohD) was identified at 12 h in LPS*_Xcc_*-treated WT, and at early and later time points in LPS*_Pst_*- and LPS*_Xcc_*-treated *bak1-4* (Table 1 and Table S1). As shown in Figure 5A, RbohD was not identified in *lbr2-2* but accumulated in WT and *bak1-4*. In addition, a putative respiratory burst oxidase homolog protein J (RbohJ) was identified only at 12 h in LPS*_Xcc_*-treated WT. RbohD and RbohF have been shown to mediate ROS accumulation in the plant during the defence response against avirulent *Pst* bacteria [145]. During plant defence against pathogens, RbohD-dependent ROS production has been shown to contribute to autophagy and hypersensitive cell death in Arabidopsis [146]. Following successful pathogen recognition, ROS production or oxidative burst is one of the earliest cellular responses [147]. These ROS regulate plant defence causing strengthening host cell walls via crosslinking of cell-wall glycoproteins, direct killing of bacteria, or act as signals to induce further defences [147,148,149]. Under biotic and abiotic stresses, heat shock proteins (HSPs) as molecular chaperons enhance plant immunity through the regulation of antioxidant enzymes system as well as the accumulation and stabilisation of PR proteins [64]. In this study, HSP70 family proteins such as heat shock 70 kDa protein 14 and heat shock 70 kDa protein 3 were identified in both LPS chemotypes-treated WT (at 12 h and 18 h in LPS*_Pst_* treatments and at 18 h in LPS*_Xcc_* treatment) and *lbr2-2* (at early and later LPS*_Pst_*- and LPS*_Xcc_* treatments), respectively (Table 1 and Table S1). Moreover, HSP90 family, such as heat shock protein 90-2 was identified at 24 h in LPS*_Pst_*-treated *bak1-4*, while heat shock protein 90-3 was identified in both LPS chemotypes-treated WT and *lbr2-2*. Figure 5B further shows that heat shock protein 90-3 was not identified in *bak1-4* but accumulated in WT and *lbr2-2*. HSP90 and HSP70 have been shown to play an essential role in the defence response against *Pseudomonas cichorii* in *N. benthamiana* [150]. Plants have evolved robust defence mechanisms against pathogen infection, during which resistance (R) genes encode R proteins that recognise pathogens and trigger defence responses. Here, disease resistance protein RPP8 was identified at 12 h in LPS*_Pst_*-treated WT, and at early and later LPS*_Pst_*- and LPS*_Xcc_*-treated *lbr2-2* (Table 1 and Table S1). Furthermore, probable disease resistance RPP8-like protein 4 was identified at later time points in LPS*_Xcc_*-treated WT, while disease resistance RPP13-like protein 4 was identified at early LPS*_Pst_*- and LPS*_Xcc_*-treated *bak1-4*. Mohr et al. [151] showed that the Arabidopsis *RPP8* gene is induced by the downy mildew pathogen *Hyaloperonospora arabidopsidis* (*Hpa*) and exogenous salicylic acid. A protein suppressor of npr1-1 constitutive 4 (SNC4) was identified at 12 h in LPS*_Pst_*-treated WT and at later LPS*_Pst_*-treated *bak1-4* as well as at early LPS*_Xcc_* treatment (Table 1 and Table S1). Figure 5C shows that SNC4 was not identified in *lbr2-2* but was accumulated in LPS*_Pst_*-treated WT and both LPS-chemotype-treated *bak1-4*. Arabidopsis snc4-1D gain-of-function mutant expressed defence marker genes *PR-1*, *PR-2* and *PDF1.2* (*plant defensin 1.2*), with enhanced disease resistance to *Hpa* Noco2 [65]. Suppressor of npr1-1 constitutive (SNC1) has been previously shown to activate the downstream defence signal transduction in both SA-dependent and SA-independent manner [152]. Hypersensitive-induced response protein 1 was identified at early and later time points in both LPS*_Pst_*- and LPS*_Xcc_*-treated *bak1-4* (Table 1 and Table S1). Hypersensitive-induced response protein 1 was not identified in WT and *lbr2-2* but was accumulated in *bak1-4* (Figure 5D). Proteins involved in the HR response are critical in the regulation of hypersensitive cell death and its associated SAR that leads to the protection of both the host plant and adjacent cells to become responsive to the evading pathogens [66,153].

### 4.4. PM-Associated Proteins Related to Metabolic Processes

Ubiquitin-26S proteasome system (UPS) selectively degrades the protein components of defence signalling, which are no more needed or are abnormal and negatively or positively regulate plant responses to a given stimulus [68]. Several LPS-responsive proteins involved in UPS were identified in this study (Table 1 and Table S1). For instance, E3 ubiquitin-protein ligase UPL 3 (at early LPS*_Pst_*- and LPS*_Xcc_* treatments), E3 ubiquitin-protein ligase RNF170-like protein (DUF 1232) (at early and later LPS*_Pst_*- and LPS*_Xcc_* treatments) and ubiquitin-activating enzyme E11 (at 12 h in LPS*_Pst_* treatment and at 18 h LPS*_Xcc_* treatment) were identified in *bak1-4*. On the other hand, polyubiquitin 3 was identified at 24 h in LPS*_Pst_*-treated WT as well as at 12 h in LPS*_Xcc_*-treated *lbr2-2*. Ubiquitin-conjugating enzyme 36 was identified at later time points in LPS*_Xcc_*-treated WT, while ubiquitin-NEDD8-like protein RUB1 was identified in WT and *lbr2-2* treated with both LPS chemotypes. Fifteen LPS-induced proteasome regulatory subunits were identified in this study (Table 1 and Table S1). For example, 26S proteasome non-ATPase regulatory subunit 1 homolog B was identified at 18 h in LPS*_Xcc_*-treated WT while 26S proteasome non-ATPase regulatory subunit 2 homolog A was identified in all three lines and treatments except in the LPS*_Pst_*-treated *lbr2-2*. Moreover, 26S proteasome non-ATPase regulatory subunit 3 homolog A was identified at 12 h and 24 h in the *bak1-4* treated with both LPS chemotypes. As shown in Figure 6A, 6S proteasome regulatory subunit 8 homolog A was not identified in *lbr2-2* and *bak1-4* but was accumulated in both LPS*_Pst_*- and LPS*_Xcc_*-treated WT. In addition, polyubiquitin 3 was not identified in *bak1-4* but accumulated at 12 h in LPS*_Pst_*-treated WT, and 0 h in LPS*_Pst_* and 12 h in LPS*_Xcc_*-treated *lbr2-2* (Figure 6B). Overall, UPS proteins were represented in all lines. In normal growth conditions, plants prevent constitutive immune response through the use of UPS system whereby U-box E3 ubiquitin ligases PUB12 and/or PUB13 E3 ligase degrades MTI receptors, such as FLS2 and LysM-containing receptor-like kinase 5 (LYK5) [41,154,155]. Bacterial *Pst* type III effectors have been shown to target proteasome subunits and suppress plant immune response against the pathogens [156]. These studies and our findings suggest a possible involvement of the UPS system in the regulation of the LPS-responsive proteins in Arabidopsis.

Cytochrome P450s are a class of oxidoreductase enzymes involved in NADPH- and/or O_2_-dependent hydroxylation, detoxification of xenobiotics, and biosynthesis phytohormones and secondary metabolites [157]. Studies have shown that 5 Arabidopsis Cytochrome P450s including CYP79B2, CYP79B3, CYP71A12, CYP71A13 and CYP71B15 participate in different steps of camalexin biosynthesis [158,159,160]. Cytochrome P450s have been shown to catalyse jasmonic acid (JA) as well as abscisic acid (ABA) biosynthesis pathways that regulate salt tolerance and wound-induced defence against biotic attacks [158,161,162]. In this study, cytochrome P450 71B6 was identified in both LPS chemotypes-treated *lbr2-2* (at early and later LPS*_Pst_* treatments and at early LPS*_Xcc_* treatments). Cyclophilins (CYPs) roles in abiotic stress responses, including protein folding, are well established, but little is known about their roles in biotic stress responses [163]. Nevertheless, these peptidyl-prolyl-cis/trans-isomerases (PPIases or immunophilins) have been linked to defence against *Xcc* infection in Arabidopsis and are suggested to be part of the immune system in plants [164]. Pogorelko et al. [165] have shown the involvement of three Arabidopsis immunophilin genes *AtCYP19*, *AtCYP57* and *AtFKBP65* in response to *Pst* infection. Several LPS-induced CYPs were identified in this study (Table 1 and Table S1). Whereas peptidyl-prolyl cis-trans isomerase CYP18-4 was identified at 18 h in LPS*_Pst_*- and LPS*_Xcc_*-treated *bak1-4*, peptidyl-prolyl cis-trans isomerase CYP19-2 was identified at 12 h in LPS*_Xcc_*-treated WT and at 18 h in LPS*_Xcc_*-treated *lbr2-2*. Figure 6C shows there was an accumulation of peptidyl-prolyl cis-trans isomerase CYP19-2 in the above stated time points but it was not identified in the *bak1-4*. On the other hand, peptidyl-prolyl cis-trans isomerase CYP21-4 was identified at early and later time points in WT in response to treatment with both LPS chemotypes. A calcium-dependent lipid-binding (CaLB domain) phosphoribosyltransferase family protein was identified in WT (at 12 h and 18 h in LPS*_Pst_* treatments, and at later LPS*_Xcc_* treatments) and *bak1-4* (early and later LPS*_Pst_*- and LPS*_Xcc_* treatments) (Table 1 and Table S1). CaLB domain or C2 domain proteins are of interest due to their involvement in signal transduction, vesicle trafficking and other cellular processes, including biotic and abiotic [166,167,168]. Arabidopsis calcium-dependent lipid-binding protein (AtCLB) has been shown to bind to the membrane lipid ceramide and regulate abiotic stress responses [168].

Secondary metabolite biosynthetic enzymes such as caffeoyl-coenzyme A (CoA) *O*-methyltransferase 1, caffeoylshikimate esterase, cinnamyl alcohol dehydrogenase 7 and flavone 3′-O-methyltransferase 1 were identified in this study (Table 1 and Table S1). Caffeoyl-CoA *O*-methyltransferase 1 was identified at 12 h in LPS*_Pst_*-treated *lbr2-2,* while caffeoylshikimate esterase was identified in LPS*_Pst_*-treated WT (at 24 h treatment) and *bak1-4* (at 18 h treatment). Caffeoyl-CoA *O*-methyltransferase 1 and caffeic acid *O*-methyltransferase 1 have been reported to be involved in the biosynthesis of defence-related secondary metabolites such as lignin, flavonoids and sinapoyl malate in Arabidopsis [169]. Arabidopsis caffeoylshikimate esterase plays a central role in the lignin biosynthetic pathway [170]. Cinnamyl alcohol dehydrogenase 7 was identified in both LPS chemotypes-treated *lbr2-2* and *bak1-4.* It was, however, not identified in the WT but showed accumulation in *lbr2-2* and *bak1-4* (Figure 6D). This enzyme is part of the Arabidopsis cinnamyl alcohol dehydrogenase multi-enzyme family that participates in lignification by catalysing the NADPH-dependent reduction in *p*-coumaroyl, caffeoyl, coniferyl, 5-hydroxyconiferyl, and sinapoyl aldehydes to their corresponding alcohols [70]. Flavone 3′-*O*-methyltransferase 1 identified at 12 h in LPS*_Pst_*-treated *lbr2-2* is involved in flavonol and lignin biosynthesis process needed for ultraviolet (UV) radiation protection, herbivore feeding deterrent and defence against pathogens [171]. These results suggest that LPS*_Pst_* and LPS*_Xcc_* triggered the synthesis of enzymes that catalyse the accumulation of defence-related proteins and secondary metabolites [172], in response to bacterial pathogen attacks.

## 5. Conclusions

In a ligand–receptor type of interaction, plants use PRRs to recognise MAMPs, leading to the activation of defence responses against pathogens. Although the mechanism of the perception of LPS and induced defence responses in mammals are well known, the mechanism in plants is unclear. The identification and understanding of PM-associated proteins involved in the perception of LPS and the induction of defence signalling in Arabidopsis WT and mutants (*lbr2-2* and *bak1-4*) will contribute to elucidation of the perception mechanism underlying the associated defence response in plants to this MAMP.

Here, label-free LC-MS/MS proteomics were used to analyse PM-associated protein fractions from Arabidopsis WT and mutants (*lbr2-2* and *bak1-4*) treated with LPS from *Pst* and *Xcc*. The study led to the identification of both common and distinct LPS-responsive PM-associated proteins in WT and mutants. These identified proteins were mainly involved in ‘perception and signalling’, ‘membrane transport and trafficking’, ‘defence and stress responses’, ‘metabolic processes’, and others. For ‘perception and signalling’ several protein families differentially identified include LRR-RLKs, LRR-RLPs, LysM-RLKs, G-type Lec S-RLK, L-type Lec-RLK, cysteine-rich RLKs, CDPKs, RLCKs PBL and BSK. Selected proteins involved in ‘membrane transport and trafficking’ include various ABC transporters, aquaporins, exocyst complex components, RABs, clathrin family proteins and flotillins. Proteins involved in ‘stress and defence’ include RbohD, RbohJ, HSP families, RPP8 and SNC4. Finally, identified proteins involved in ‘metabolic processes’ were those belonging to the ubiquitin and proteasome family, as well as CYP proteins, calcium-dependent lipid-binding (CaLB domain) phosphoribosyltransferase family protein, caffeoyl-CoA O-methyltransfersase 1, caffeoylshikimimate esterase, cinnamyl alcohol dehydrogenases and flavone 3′-O-methyltransferase 1.

It is interesting to note that using this label-free LC-MS/MS proteomics approach (with focus on identification but not quantification of proteins), BAK1 was identified in both LPS*_Pst_*- and LPS*_Xcc_*-treated WT and *lbr2-2* above the significance threshold scores. This was, however, not included in the discussion for selected proteins since BAK1 was also detected in the MgCl_2_-treated 24 h control (also above the scores). It should nonetheless be said that had a quantitative approach been followed, a similar trend may have been observed when comparing the total peak intensities (Figure S10) from the current data. Here, the LPS*_Pst_*-treated WT sample at 24 h showed an increase in the intensity of BAK1 compared to the control as is also seen for the LPS*_Xcc_*-treated WT over the study period, except at the 12 h point. Furthermore, the LPS*_Pst_*-treated *lbr2-2* intensity peaked at the 12 h point, well above that of the control, while the LPS*_Xcc_*-treated *lbr2-2* showed a steady increase over the treatment period, *albeit* below that of the control. The differences across the time points could also hint toward a dynamic endocytosis process and could be substantiated by the identification of clathrin family proteins in all three lines for both chemotypes.

Overall, LPS*_Pst_* and LPS*_Xcc_* chemotypes (representing penta- and hexaacylated lipid A moieties, respectively) resulted in common but, importantly, also distinct PM and associated LPS-responsive proteins in the WT and mutant plants. These findings suggest that different LPS chemotypes are most likely trigger related but with distinctive responses due to their chemical composition and configuration. Moreover, since the mechanism of the LPS perception determines the protein expression profiles, the nuanced differences observed in WT *vs*. mutants as well as *lbr2-2 vs*. *bak1-4* could be linked to a possible involvement of LBR2 and BAK1 in LPS-induced MTI and distinct downstream signalling, *albeit* with some commonality where the loss-of-function is probably compensated for by alternative proteins that still trigger a response subsequent to perception within a receptor complex at the PM. Based on the type of RLKs and RLPs identified (especially those that are involved in other bacterial and fungal MAMP perception and signalling), we propose the participation of multiple receptors (e.g., carbohydrate- and lipid specific) and/or co-receptors in the recognition of the tripartite LPS lipoglycans and resultant signalling in Arabidopsis.

## Figures and Tables

**Table 1:** Summary of the selected LPS-responsive PM-associated proteins (^#^) from Arabidopsis WT, *lbr2-2* and *bak1-4* plants treated with LPS from *Pst* and *Xcc*. Grey shading indicates proteins not identified in specific line(s) and/or treatment according to significance threshold scores.

Protein	WT (*Pst*)	WT (*Xcc*)	*lbr2-2* (*Pst*)	*lbr2-2* (*Xcc*)	*bak1-4* (*Pst*)	*bak1-4* (*Xcc*)
**Perception and Signalling**						
SERK2	X					
BKK1					X	
BIR1			X	X		
BIR2			X			
BIR3			X	X	X	X
RLP23			X	X	X	X
RLP51					X	X
SOBIR1	X	X				
CERK1				X	X	X
LYK3				X	X	
LYK4					X	
G-type Lec S-RLK family			X			X
L-type Lec-RK S.1		X	X		X	
L-type Lec-RK IV.4					X	X
L-type Lec-RK VII.1	X					
Cysteine-rick RLK family	X	X	X	X	X	X
CDPK family	X	X	X	X	X	X
PBL family	X PBL27	X PBL27	X PBL22	X PBL22		X PBL1
BSK family	X	X			X	X
**Membrane Transport and Trafficking**						
ABC transporter family	X	X	X	X	X	X
Aquaporin TIP1-2					X	X
Aquaporin PIP1-3	X	X	X	X		
Aquaporin PIP2-3	X	X		X	X	
Aquaporin PIP1-4			X	X	X	X
RAB family	X RABC, RABG, RABF	X RABC, RABG, RABF	X RABB, RABC, RABG	X RABB, RABC, RABG	X RABF	X RABF
Syntaxin					X	X
Exocyst complex component	X	X	X	X	X	X
Flotillin	X	X	X	X		
Clathrin family	X	X	X	X	X	X
**Stress and Defence**						
RbohD		X			X	X
RbhoJ		X				
HSP family	X	X	X	X	X	X
RPP8	X		X	X		
RPP13					X	X
SNC4	X				X	X
Hypersensitive-induced response protein 1					X	X
**Metabolic Processes**						
UPL					X	X
Ubiquitin family	X	X	X	X	X	X
Proteasome family	X	X	X	X	X	X
CYPs	X	X	X	X	X	X
CaLB domain protein	X	X			X	X
Caffeoyl-CoA O-methyltransfersase 1			X			
Caffeoylshikimate esterases	X				X	
Cinnamyl alcohol dehydrogenases			X	X	X	X
Flavone 3′-O-methyltransferase 1			X			

^#^ For contextual perspective of the identified LPS-responsive proteins in signalling cascades, the reader is referred to Figure S9 (taken from [2]).

## Data Availability

Restrictions apply to the availability of raw data. Data were obtained from CPGR, South Africa, and are available from the corresponding author (for data set identifier, username and password) for repository JPST001598/PXD034031. Repository citation: Okuda, S. et al. jPOSTrepo: an international standard data repository for proteomes. Nucl. Acids Res. 45 (D1): D1107-D1111 (2017). doi: 10.1093/nar/gkw1080.

## Supplementary Materials

The following supporting information can be downloaded at: https://www.mdpi.com/article/10.3390/membranes12060606/s1, Figure S1: Representative characterisation of Arabidopsis PM fractions by Western blot analysis; Figure S2: Summary of the Byonic™ software data analysis for Arabidopsis PM-associated protein identification; Figure S3: The protein–protein interaction networks of PM-associated proteins identified from LPS_*Pst*_-treated Arabidopsis WT; Figure S4: The protein–protein interaction networks of PM-associated proteins identified from LPS_*Xcc*_-treated Arabidopsis WT; Figure S5: The protein–protein interaction networks of PM-associated proteins identified from LPS_*Pst*_-treated Arabidopsis *lbr2-2*; Figure S6: The protein–protein interaction networks of PM-associated proteins identified from LPS_*Xcc*_-treated Arabidopsis *lbr2-2*; Figure S7: The protein–protein interaction networks of PM-associated proteins identified from LPS_*Pst*_-treated Arabidopsis *bak1-4*; Figure S8: The protein–protein interaction networks of PM-associated proteins identified from LPS_*Xcc*_-treated Arabidopsis *bak1-4*; Figure S9: Generalised view of MAMP perception via PRRs, illustrating the potential impact of knockout (KO) effects that influence MAMP-triggered immunity; Figure S10: Analysis of total peak intensity of PM-associated BAK1 detected in the control, LPS_*Pst*_- and LPS_*Xcc*_-treated WT and *lbr2-2* within a 24 h period. Table S1: LPS-responsive PM-associated proteins identified from the Arabidopsis WT, *lbr2-2* and *bak1-4* plants treated with LPS from *Pst* and *Xcc*.
