# Supplementary material for: Plasma Membrane-Associated Proteins Identified in Arabidopsis Wild Type, lbr2-2 and bak1-4 Mutants Treated with LPSs from Pseudomonas syringae and Xanthomonas campestris
Source: Membranes (Basel). 2022 Jun 10;12(6):606. doi: 10.3390/membranes12060606 (PMC9230897; doi:10.3390/membranes12060606)
Supplement: Supplementary file 1 [file membranes-12-00606-s001.zip › membranes-1761648-supplementary.pdf]

membranes

MDPI

Supplementary materials

Plasma Membrane-Associated Proteins Identified in Arabidopsis Wild Type, *lbr2-2* and *bak1-4* Mutants Treated with LPSs from *Pseudomonas syringae* and *Xanthomonas campestris*

Benedict C. Offor, Msizi I. Mhlongo, Ian A. Dubery and Lizelle A. Piater *

Department of Biochemistry, University of Johannesburg, Auckland Park, Johannesburg 2006, South Africa; benedictoffor@gmail.com (B.C.O.); mmhlongo@uj.ac.za (M.I.M.); idubery@uj.ac.za (I.A.D.)

* Correspondence: lpiater@uj.ac.za; Tel.: +27-11-559-2403

Relevant Figures and Tables referred to but not included in the main text are provided in this supplementary file. Restrictions apply to the availability of raw data. Data were obtained from CPGR, South Africa, and are available from the corresponding author (for data set identifier, username and password) for repository JPST001598/PXD034031. Repository citation: Okuda, S. et 521 al. jPOSTrepo: an international standard data repository for proteomes. Nucl. Acids Res. 45 (D1): 522 D1107-D1111 (2017). doi: 10.1093/nar/gkw1080.

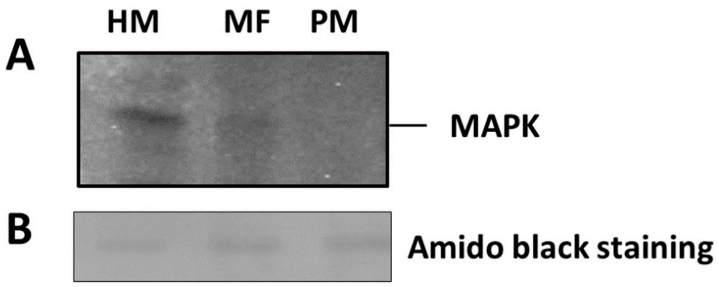

Figure S1. Representative characterisation of Arabidopsis PM fractions by Western blot analysis. **(A)** MAPK was probed with anti-active MAPK pAb, rabbit (pTEpY) antibody in the isolated Arabidopsis homogenate (HM), microsomal fraction (MF) and plasma membrane (PM-associated) subsequent to 12 h LPS from *Pst* treatment. **(B)** Amido Black PVDF nitrocellulose membrane-stained loading control indicates that lack of MAPK activity in PM fraction is not due to the absence or unequal loading of proteins.

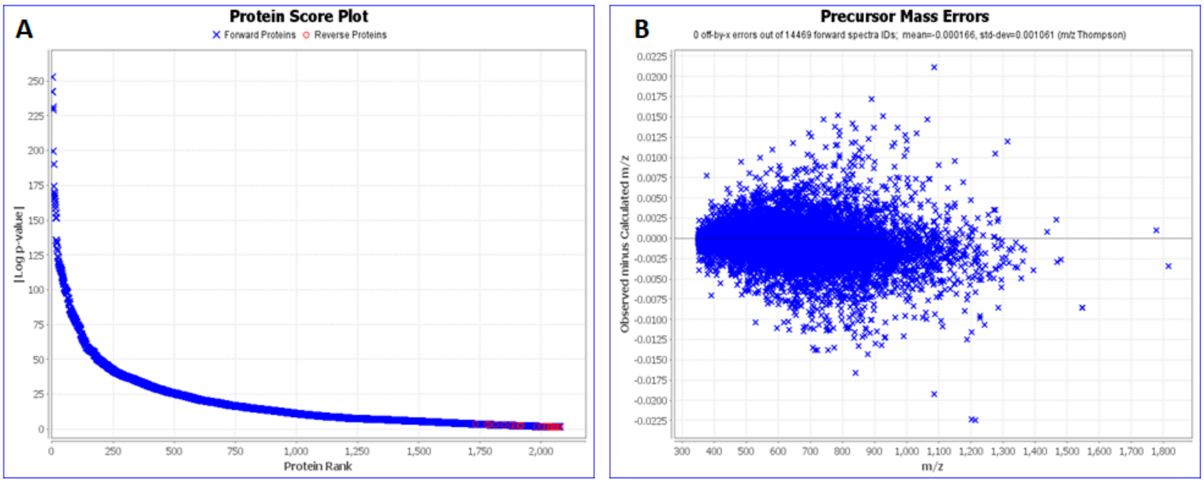

Figure S2. Summary of the Byonic™ software data analysis for Arabidopsis PM-associated protein identification. (**A**) A score plot representation of the differential abundance of proteins. (**B**) Representation of the precursor mass error loadings plot. This indicates the difference between the calculated mass and the observed mass of the peptides.

Table S1. LPS-responsive PM-associated proteins identified from the Arabidopsis WT, *lbr2-2* and *bak1-4* plants treated with LPS from *Pst* and *Xcc*. Proteins identified in the control ($MgCl_2$-treated) were not selected as significant proteins. LPS_{Pst} and LPS_{Xcc}-responsive proteins were deemed significant over the treatment period (0, 12, 18 and 24 h) with a Byonic™ score ≥ 300 and log probability ≥ 1. Selected proteins with the functional categories such as membrane transport and trafficking, metabolic process, perception and signalling, and stress and defence response are coloured (only WT: Green; WT and *lbr2-2*: Pink; WT and *bak1-4*: Yellow; only mutants: Blue; all 3 lines: Grey and with a lighter shade for only 1 chemotype). The proteins indicated in bold are discussed in the main text.

Protein ID	Uniprot ID	|Log prob|	Byonic™ score	WT (h)		*lbr2-2* (h)		*bak1-4* (h)	
				Pst	*Xcc*	*Pst*	*Xcc*	*Pst*	*Xcc*
**Membrane Transport and Trafficking**									
**ABC transporter B family member 17**	**Q9LSJ6**	3.04	335.7			18			
**ABC transporter B family member 20**	Q9M3B9	8.94	442.2					0, 18	
**ABC transporter B family member 21**	Q9M1Q9	7.31	331.5	18	12, 18				
**ABC transporter B family member 22**	Q9LSJ2	1.92	304.4				24		
**ABC transporter B family member 29 chloroplastic**	Q9LZB8	4.94	376.5			12, 18			
**ABC transporter B family member 4**	O80725	7.88	333.3	24	12, 18				24
**ABC transporter B family member 6**	Q8LPT1	4.10	317.5		24			24	0, 12, 18
**ABC transporter C family member 4**	Q7DM58	5.92	340.4					0, 18	0, 18
**ABC transporter F family member 1**	Q9FJH6	6.89	451.0						0
**ABC transporter G family member 12**	Q9C8K2	4.85	344.3			12			

**ABC transporter G family member 32**	O81016	6.53	336.4	12	12, 18				
Acyl-CoA-binding domain-containing protein 2	Q9STP8	4.06	303.0	18, 24		12	0, 24		
Acyl-CoA-binding domain-containing protein 6	P57752	4.63	327.2	18, 24	0, 18				
ADPATP carrier protein 3 mitochondrial	O49447	5.74	312.4		0	0	0, 18, 24		
ADP-ribosylation factor A1F	Q6ID97	8.03	521.9	0, 18, 24	0, 18, 24				
ALA-interacting subunit 1	Q9LTW0	4.05	315.7	0			12, 24	12, 18, 24	0, 12, 18, 24
Amino acid permease 1	Q42400	6.79	300.4						18
Amino acid transporter AVT1C	F4IUW3	6.83	346.4		12				
Ammonium transporter 2	Q9M6N7	4.94	368.8						12
AP-2 complex subunit alpha-1	Q8LPL6	4.10	300.4				0		
AP-2 complex subunit alpha-2	Q8LPK4	4.10	300.4				0	0, 18, 24	0, 12
**Aquaporin PIP1-1**	P61837	12.94	701.0					0	0, 18
**Aquaporin PIP1-3**	Q08733	6.15	312.5	24	12, 18	0, 12, 24	18, 24		
**Aquaporin PIP2-3**	P30302	6.84	363.2	12, 18	0, 18		18, 24	12, 18	
**Aquaporin TIP1-2**	Q41963	14.91	808.9					0, 12, 18, 24	0, 12, 18, 24
AT2G33120 protein	B9DH97	4.16	403.7					0, 18	0
ATP synthase subunit beta-3 mitochondrial	Q9C5A9	10.05	628.6	0, 12, 18, 24	0, 12, 18, 24				
Auxin efflux carrier component 4	Q8RWZ6	7.85	329.8		18	0			
Auxin efflux carrier component 7	Q940Y5	6.38	333.4		18	0, 12		0, 12, 18	0, 12, 18, 24
Auxin transporter protein 1	Q96247	6.60	423.8	12, 18	12, 18				
Auxin transporter-like protein 1	Q9LFB2	8.76	452.9			0, 24		0, 18	0, 18
Bet1-like SNARE 1-1	Q9M2J9	6.49	397.1				24		
Beta-adaptin-like protein	F4JNZ8	6.04	347.7		24	12	18	0	0, 18
Calcium-transporting ATPase 1 endoplasmic reticulum-type	P92939	8.22	432.8			0, 12, 18, 24	0, 18, 24		
Calcium-transporting ATPase 4 endoplasmic reticulum-type	Q9XES1	7.75	463.9		0, 12, 18			12	0, 18, 24
Calcium-transporting ATPase 9 plasma membrane-type	Q9LU41	5.85	340.8				24		
**Clathrin heavy chain 1**	Q0WNJ6	5.75	360.3	12	18, 24			0, 12, 18, 24	12, 24
**Clathrin heavy chain 2**	Q0WLB5	8.94	560.5						24
Clathrin interactor EPSIN 2	Q67YI9	7.03	341.0			24	0, 24		
**Clathrin light chain 1**	Q9SKU1	6.34	391.3			0, 12, 24	0, 24		
**Clathrin light chain 2**	O04209	8.94	487.7	12	24				
Coatomer subunit beta-1	Q9SV21	7.53	327.5	0, 24	0, 12, 18, 24	12	0, 24		
Coatomer subunit beta-2	Q9SV20	8.26	473.5					0, 18, 24	12, 18, 24

Coatomer subunit delta	Q93Y22	7.00	341.5	12	12, 18, 24	0, 12, 24	0		
Coatomer subunit gamma	Q0WW26	6.48	363.3	0, 18, 24	0, 12, 18, 24				
Coatomer subunit zeta-3	Q8H1F4	4.90	391.4			0, 12, 24			
Dynamin-2A	Q9SE83	6.58	516.5			24	0, 12, 18		
Equilibrative nucleotide transporter 1	Q8VXY7	6.87	311.2	0, 12	0, 18, 24	0, 12, 24	0		
**Exocyst complex component EXO70A1**	Q9LZD3	6.74	325.5		18	12			0
**Exocyst complex component EXO70B1**	Q9FGH9	8.16	409.2					12	0
**Exocyst complex component SEC15B**	F4JHH5	5.95	365.1						0
**Exocyst complex component SEC3A**	Q9SX85	7.01	312.7					0, 12	0, 12
**Exocyst complex component SEC5A**	Q8S3U9	6.31	429.3			0, 12	0	12, 18	0, 12
**Exocyst complex component SEC6**	Q94AI6	6.34	344.0	12	12, 24	0, 12	0, 24		
**Exocyst complex component SEC8**	Q93YU5	4.60	323.3			0, 12	0	0, 12	0, 12, 24
**Flotillin-like protein 1**	Q501E6	6.29	405.8	12, 18, 24	24	0, 12, 24	0, 18, 24		
**Flotillin-like protein 2**	Q4V3D6	6.10	363.8	0, 12, 24	24				
Gamma-soluble NSF attachment protein	Q9SPE5	7.60	418.2	12, 18, 24	0, 12, 24	0, 18			
GTP-binding protein SAR1A	O04834	7.48	437.8	18	18	12, 18, 24	0		
High-affinity nitrate transporter 3.1	Q9FGS5	9.25	389.4					12	12
Lysine histidine transporter 1	Q9FKS8	4.53	300.8					0, 12	12, 18
Mechanosensitive ion channel protein 10	Q9LYG9	7.25	334.5					0	
Membrane magnesium transporter	Q2HIM5	3.72	318.7			0, 12	0	18	0
Mitochondrial pyruvate carrier 1	Q949R9	6.84	473.7					18	0, 18
Monosaccharide-sensing protein 2	Q8LPQ8	8.63	410.6		12			0, 12, 18, 24	0, 12, 18, 24
Novel plant SNARE 12	Q9LNH6	3.90	382.4			12			
Novel plant SNARE 13	Q9LRP1	6.44	398.9						18
Nucleobase-ascorbate transporter 3	Q8GZD4	5.26	367.4					0, 12, 18, 24	0, 12, 18, 24
Nucleobase-ascorbate transporter 7	Q0WPE9	7.22	358.0		12				
Oligopeptide transporter 3	O23482	7.15	357.1			0, 12	18		
Oxysterol-binding protein-related protein 3A	Q9LZM1	6.77	311.8			0			
Patellin-2	Q56ZI2	7.31	308.4			0, 12, 24	0, 18, 24		
Patellin-3	Q56Z59	6.30	373.3	0, 12, 24	0, 18, 24	0, 12, 24	0, 18		
Patellin-4	Q94C59	6.95	436.8					0, 18, 24	0, 18

Phosphatidylinositol/phosphatidylcholine transfer protein SFH5	Q8GXC6	5.85	374.6						0
Phospholipid-transporting ATPase 1	P98204	6.45	353.9		18		0, 24	0, 12, 18	0, 12, 18
Phospholipid-transporting ATPase 3	Q9XIE6	6.33	391.9					0, 12, 18	0, 18, 24
Plasma membrane intrinsic protein 1B	A8MRW1	8.58	488.7			0, 12, 18, 24	0, 12, 18, 24		
Potassium transporter 13	Q8LPL8	8.54	515.8	12	12, 18				
Potassium transporter 4	Q9LD18	6.52	372.3					0	0, 12
Potassium transporter 8	Q9M7J9	4.74	328.2						0
**Probable aquaporin PIP1-4**	Q39196	6.18	330.7			0, 12, 24	0, 24	0, 12, 18, 24	0, 12, 18
Probable clathrin assembly protein At4g32285	Q8S9J8	3.25	321.4				18		
Probable cyclic nucleotide-gated ion channel 6	O82226	6.39	336.6					12	
Probable phospholipid-transporting ATPase 11	Q9SAF5	3.98	316.3			12	24		
Probable purine permease 18	Q9C508	9.36	462.8					12, 18	
Probable voltage-gated potassium channel subunit beta	O23016	8.59	490.5					18	0
Protein NRT1/ PTR FAMILY 6.3	Q05085	9.07	434.2					18	
Protein NRT1/ PTR FAMILY 8.1	Q9M390	8.18	537.8			0, 18	0, 12, 24		
Protein OSCA1	Q9XEA1	5.82	401.5			0	0		
Protein TPLATE	F4J8D3	6.54	352.0					0, 12	0, 12, 24
**Protein transport protein Sec61 subunit beta**	Q9FKK1	8.66	377.3					18	
Protein YIPF	Q93VH1	5.81	339.2				24		
Putative GTP-binding protein ara-3	Q9FJF1	8.91	592.2					12, 18	
Putative vesicle-associated membrane protein 726	Q9MAS5	6.44	550.8			0		24	0
Rac-like GTP-binding protein ARAC3	Q38912	5.68	335.9	0		12		0	
Rac-like GTP-binding protein ARAC4	Q38919	5.68	335.9	0, 24	24	12			
Rac-like GTP-binding protein ARAC5	Q38937	5.68	335.9	0, 24		12			
**Ras-related protein RABA1a**	P28185	7.04	494.7		18				
**Ras-related protein RABA1d**	Q9SN35	6.90	411.9	12, 24	12, 24			0, 12, 18	24
**Ras-related protein RABA1f**	Q9FJH0	7.43	523.1					0, 12	18
**Ras-related protein RABA1g**	Q9LK99	4.53	354.0					0, 18	12
**Ras-related protein RABA2a**	O04486	7.23	302.3	24	18, 24		0		
**Ras-related protein RABA2b**	Q9LNW1	6.72	483.1			18			

**Ras-related protein RABA2c**	Q96283	6.96	468.7			0, 12, 24	18, 24		
**Ras-related protein RABA2d**	Q9FIF9	6.96	468.7			18	12		
**Ras-related protein RABA4a**	Q9FJN8	6.33	417.9					0, 12, 18, 24	0, 12, 18, 24
**Ras-related protein RABA4b**	Q9SMQ6	7.09	310.4	24	0	0, 12, 18	0		
**Ras-related protein RABA5a**	Q9FGK5	4.09	400.1			0			
**Ras-related protein RABA5b**	Q9SRS5	4.11	317.8			0			
**Ras-related protein RABA5c**	P28187	4.11	317.8			0			
**Ras-related protein RABB1c**	P92963	8.23	475.2			0, 12, 18, 24	0, 12, 18, 24		
**Ras-related protein RABC1**	O23657	6.32	357.1	0, 18, 24	12, 24	24	18, 24		
**Ras-related protein RABD1**	Q9ZRE2	12.33	668.8					18	
**Ras-related protein RABD2a**	P28188	8.01	437.1	0, 12, 24	18, 24			0	
**Ras-related protein RABD2b**	Q9FPJ4	8.73	526.1			12, 18, 24	0, 18, 24		
**Ras-related protein RABD2c**	Q9SEH3	9.11	574.2	12	0			0, 12, 18	12, 18, 24
**Ras-related protein RABE1a**	O24466	5.23	309.6	0, 24	18, 24	12, 24	0, 12, 24		0
**Ras-related protein RABE1c**	P28186	6.24	349.8			18	12		
**Ras-related protein RABE1d**	Q9LZD4	8.17	456.5			0, 12, 24	0, 18, 24	12	
**Ras-related protein RABF1**	Q9CB01	8.20	498.3					0, 18	0, 18
**Ras-related protein RABF2b**	Q9SN68	6.75	341.8	24	0, 12, 18, 24			0, 18	12, 18
**Ras-related protein RABG1**	Q948K6	6.38	415.5	24	0, 12, 18, 24	12, 18, 24	0, 12, 18, 24		
**Ras-related protein RABG3b**	O04157	7.06	340.6	0, 12, 24	12, 24		0, 18		
**Ras-related protein RABG3e**	Q9XI98	6.39	312.0	12, 24	18	0, 12, 24	0, 12		
Root phototropism protein 3	Q9FMF5	5.92	377.8					0	
S-adenosylmethionine carrier 1 chloroplastic/mitochondrial	Q94AG6	7.43	334.8	12	0, 18, 24	0, 12, 24	0, 18		
**SEC6**	F4IA34	8.33	440.8					0	12
Secretory carrier-associated membrane protein	F4HTI7	7.46	434.6	0, 18, 24	0, 12, 18				
SecY protein transport family protein	Q8RWJ5	10.58	612.0					0, 12, 18, 24	0, 12, 18, 24
Sodium/hydrogen exchanger 1	Q68KI4	8.34	473.9					0, 12, 18	0, 18
Sorting nexin 1	Q9FG38	2.08	332.1					18, 24	
Sucrose transport protein SUC1	Q39232	4.93	309.8	12					
Sucrose transport protein SUC1	Q39232	5.02	316.4			12			
Sugar transport protein 4	Q39228	4.70	354.4					12	12
**Syntaxin-122**	Q9SVC2	6.59	377.1					12, 18	0, 12, 18, 24
Thylakoid ADPATP carrier protein chloroplastic	Q9M024	6.79	456.7					0, 12, 18, 24	0, 18, 24
TOM1-like protein 1	Q9LFL3	4.24	300.4	12, 18					
Transducin/WD40 repeat-like superfamily protein	F4JN30	1.90	309.2	0				0	0

Vacuolar protein sorting-associated protein 32 homolog 1	O82197	3.55	317.3				24		
Vacuolar protein sorting-associated protein 32 homolog 2	Q9SZE4	3.55	317.3				24		
Vacuolar sorting protein 39	Q8L5Y0	7.56	427.3						0
Vacuolar-sorting receptor 1	P93026	6.11	340.3			12			
VAMP-like protein YKT61	Q9ZRD6	4.22	337.1		0	0, 18	0, 18, 24		
Vesicle transport v-SNARE 12	Q9SEL5	3.41	303.6			12			
Vesicle-associated membrane protein 721	Q9ZTW3	7.26	581.1	18					18
Vesicle-associated membrane protein 722	P47192	4.16	403.7					0, 18, 24	0
Vesicle-associated membrane protein 725	O48850	6.44	550.8			0		24	0
Vesicle-associated protein 1-3	Q84WW5	7.96	360.0	12	18	12, 24			
Vesicle-associated protein 2-1	Q9LVU1	6.85	361.6	12				18	12
Vesicle-associated protein 2-2	B9DHD7	6.29	393.4			0, 12, 18, 24	0, 18, 24	0, 12, 18	0, 18
Vesicle-fusing ATPase	Q9M0Y8	4.46	305.0		18	0, 12	0	0, 24	0, 18
V-type proton ATPase subunit F	Q9ZQX4	5.94	417.1					0, 18	
V-type proton ATPase subunit G1	O82628	17.38	906.6					0, 12, 18	0, 12, 18, 24
**Metabolic Process**									
1-acyl-sn-glycerol-3-phosphate acyltransferase 1 chloroplastic	Q8GXU8	7.04	513.9	18	0, 12, 18				
1-aminocyclopropane-1-carboxylate oxidase 2	Q41931	7.59	315.7		0				
**26S proteasome non-ATPase regulatory subunit 1 homolog B**	Q9MAT0	7.03	366.4		18				
**26S proteasome non-ATPase regulatory subunit 2 homolog A**	Q9SIV2	7.58	387.3	0, 24	0, 24		0, 12	0, 12, 18, 24	0, 12, 18
**26S proteasome non-ATPase regulatory subunit 3 homolog A**	Q9LNU4	4.76	310.9			12, 24	12, 24		
**26S proteasome non-ATPase regulatory subunit 3 homolog B**	Q9LQR8	4.57	331.8	18	0, 18	0, 24			18
**26S proteasome non-ATPase regulatory subunit 4 homolog**	P55034	7.12	329.0		0		0, 18		
**26S proteasome non-ATPase regulatory subunit 6 homolog**	Q93Y35	5.67	307.2	12, 24					
**26S proteasome non-ATPase regulatory subunit 7 homolog A**	O24412	6.74	327.9			12	0, 24		

**26S proteasome regulatory subunit 7 homolog A**	Q9SSB5	4.99	370.7	12	0, 18				
**26S proteasome regulatory subunit 8 homolog A**	Q9C5U3	6.54	303.1	0, 18, 24	0, 18, 24				
**26S proteasome regulatory subunit S10B homolog B**	Q9MAK9	5.56	316.5			0, 12		0, 12, 18, 24	0, 12, 18
3-oxo-5-alpha-steroid 4-dehydrogenase (DUF1295)	F4HRY1	5.98	382.6			0, 12, 18	0, 12, 18		
5-methyltetrahydropteroyltriglutamate--homocysteine methyltransferase 2	Q9SRV5	8.53	441.7	12, 18	0, 12, 18	12, 24	0		
5'-methylthioadenosine/S-adenosylhomocysteine nucleosidase 1	Q9T0I8	5.73	304.5		24				
7-dehydrocholesterol reductase	Q9LDU6	6.37	358.5			12			
Acetyl-CoA acetyltransferase cytosolic 1	Q8S4Y1	8.16	446.2			0, 12, 24	0, 18, 24		
Acetyl-CoA carboxylase 1	Q38970	6.15	335.3						0
Acyl-CoA-binding domain-containing protein 1	Q9SM23	3.97	307.0	12	18				
Adenine nucleotide alpha hydrolases-like superfamily protein	Q94II5	7.18	386.2	12	24	0, 12, 24	0, 12	18	
Adenine phosphoribosyl transferase 1	F4HSX1	8.26	519.5						18
Adenylylsulfatase HINT1	Q8GUN2	7.37	391.6			0	24		
ADP-ribosylation factor GTPase-activating protein AGD12	Q9FVJ3	7.13	321.4	18	24	12	0		
Alanine--glyoxylate aminotransferase 2 homolog 1 mitochondrial	Q940M2	8.74	381.8			12	0		
Allene oxide cyclase 3 chloroplastic	Q9LS01	7.75	399.5					12	24
Alpha/beta-Hydrolases superfamily protein	Q8VZV6	6.40	307.5					0, 18	18
Aminopeptidase M1	Q8VZH2	6.15	310.8		24				
Aspartate aminotransferase 3 chloroplastic	P46644	5.73	376.9			0, 12, 18	0, 12, 18, 24		18
Aspartate aminotransferase cytoplasmic isozyme 1	P46645	5.34	302.1	0, 12, 18, 24	0				
Aspartate--tRNA ligase 2 cytoplasmic	Q9M084	10.73	605.9					0, 12, 18, 24	0, 12, 18
At1g29790	Q8RWB7	6.92	324.2	0	0, 12, 18	18, 24	0, 12, 24		
At4g29870	Q9SZQ8	7.35	342.3	0, 18	12, 24				0, 12
At4g33120	Q84TE2	4.04	305.5	0, 18, 24					
AT4g35220/F23E12_220	Q94JT5	4.32	353.8	12, 18, 24					

AT5g10730/MAJ23_90	Q93VH5	8.77	411.5	0, 12, 18	0, 18, 24	12, 24	0, 24		
At5g19440	Q29Q34	4.18	315.0			24			
ATP sulfurylase 1 chloroplastic	Q9LIK9	6.29	388.4			0, 12, 24	0, 18, 24	18	12, 24
ATP-citrate synthase beta chain protein 2	Q9FGX1	5.85	348.1						18
ATP-dependent helicase/deoxyribonuclease subunit B	Q8L776	7.25	316.9			0		0, 12, 24	0, 12, 24
BTB/POZ domain-containing protein At1g30440	Q9S9Q9	7.24	367.7		18		18		
**Caffeoyl-CoA O-methyltransferase 1**	O49499	3.65	301.2			12			
**Caffeoylshikimate esterase**	Q9C942	7.43	327.5	24				18	
Calcium-dependent lipid-binding (CaLB domain) plant phosphoribosyltransferase family protein	Q9C8H3	6.52	355.7	12, 18	0, 18, 24			0, 12, 18, 24	0, 12, 18
Cell division control protein 48 homolog D	Q9SCN8	9.01	495.4						12
Cell division control protein 48 homolog E	Q9LZF6	9.01	495.4						12
**Cinnamyl alcohol dehydrogenase 7**	Q02971	3.48	407.7			0, 12, 18, 24	0, 18, 24	0, 12, 18	0, 18, 24
Cullin-associated NEDD8-dissociated protein 1	Q8L5Y6	6.23	422.6					12	0
Cyclin-dependent kinase A-1	P24100	6.43	329.7					0	0
**Cytochrome P450 71B11**	P58049	4.36	329.2		0				
**Cytochrome P450 71B6**	O65787	6.35	371.3			0, 12, 18, 24	0, 12		
D-lactate dehydrogenase [cytochrome] mitochondrial	Q94AX4	7.09	430.2	0, 12, 24	0, 12, 24			0, 12, 18	0, 12, 18
**E3 ubiquitin-protein ligase RNF170-like protein (DUF 1232)**	F4I1E4	6.59	346.2					0, 12, 18	0, 12, 18, 24
**E3 ubiquitin-protein ligase UPL3**	Q6WWW4	7.68	431.6					12	0, 12
Early nodulin-like protein 2	Q9T076	5.59	323.0	0, 12, 18, 24	0, 12, 18, 24				
Eukaryotic aspartyl protease family protein	Q9ZVS4	6.25	370.3						18
Expressed protein	O48783	6.69	357.0						0
Expressed protein OS	O48783	5.30	333.4	12, 18					
F9K20.7 protein	Q9ZVA7	6.76	324.6			12			
Fasciclin-like arabinogalactan protein 1	Q9FM65	7.62	316.2	0, 12, 18, 24	0, 12, 18	0, 24	0	18	18
Flavodoxin-like quinone reductase 1	F4K0D0	6.63	365.1		24				

**Flavone 3'-O-methyltransferase 1**	Q9FK25	7.25	366.3			12			
Gamma-glutamyl peptidase 1	Q9M0A7	1.74	310.6	12	18				
General regulatory factor 9	F4IP55	6.86	457.4					18	0, 18
Glucan endo-13-beta-glucosidase 5	Q9M088	6.14	316.3	0			0		
Glutamate dehydrogenase 2	Q38946	5.73	327.0			0, 12, 24	24	0, 18	18
Glycerol-3-phosphate acyltransferase 9	Q8GWG0	6.16	366.9			0, 12, 24	0	0, 12	0, 18
Glycerophosphodiester phosphodiesterase GDPDL3	Q9SZ11	8.70	512.5			0, 12, 18, 24	0, 18, 24	0, 12, 18, 24	0, 12, 18, 24
Glycerophosphodiester phosphodiesterase GDPDL4	Q9FJ62	7.23	304.5			0	24		
Heavy metal-associated isoprenylated plant protein 26	Q9SZN7	4.98	332.6					0, 12	24
IAA-amino acid hydrolase ILR1-like 4	O04373	9.08	461.4					18	18, 24
Inositol-phosphate phosphatase	Q9M8S8	2.87	301.5	24	18, 24	24			
Isocitrate dehydrogenase [NAD] regulatory subunit 1 mitochondrial	Q8LFC0	8.72	523.8			0, 12, 24	0, 12		
Lactoylglutathione lyase GLX1	O65398	6.25	320.4	0, 24	12, 24	0, 12	0, 24		
LL-diaminopimelate aminotransferase chloroplastic	Q93ZN9	4.98	364.0					18	
Long chain acyl-CoA synthetase 4	Q9T0A0	5.68	311.5	12, 18, 24	18				
Lysophospholipid acyltransferase 1	F4IDU4	6.60	318.0	24	0, 18, 24	12, 24	0, 12, 18, 24		
Methylcrotonoyl-CoA carboxylase beta chain mitochondrial	Q9LDD8	6.13	330.8			12	0	0, 18	18
Monodehydroascorbate reductase 2	Q93WJ8	7.50	315.9			12	0		
Monogalactosyldiacylglycerol synthase 1 chloroplastic	O81770	6.74	337.6	12					
N-acylphosphatidylethanolamine synthase	Q9ZV87	6.37	311.4			0			
NAD(P)-binding Rossmann-fold superfamily protein	Q9SZB3	8.36	506.9	24	0, 18, 24			0, 12, 18, 24	0, 12, 18, 24
NADH-ubiquinone oxidoreductase chain 1 (Fragment)	Q1ZXW0	3.74	319.8	12					
Nitrate reductase [NADH] 2	P11035	6.27	373.9	0, 12	0, 18, 24	0			
Nitrilase 3	P46010	9.24	444.7		18				
Pectin acetylesterase 11	Q9FH82	6.67	364.2	12				0, 12	
Peptidase M1 family protein	F4I3R1	7.58	346.3			12			

**Peptidyl-prolyl cis-trans isomerase CYP18-4**	Q42406	8.83	519.8					18	18
**Peptidyl-prolyl cis-trans isomerase CYP19-2**	Q9SKQ0	7.90	367.7		12		18		
**Peptidyl-prolyl cis-trans isomerase CYP21-2**	Q8L8W5	4.32	322.4				0		
**Peptidyl-prolyl cis-trans isomerase CYP21-4**	Q9C835	7.46	358.1	12, 18, 24	0, 12, 18, 24				
**Peptidyl-prolyl cis-trans isomerase FKBP42**	Q9LDC0	6.93	314.0						0
Peroxisomal fatty acid beta-oxidation multifunctional protein AIM1	Q9ZPI6	4.50	324.4	0, 12	18, 24	0			
Phosphoglucomutase/phosphomannomutase family protein	F4I6W4	7.70	390.3					0, 12, 18, 24	0, 18, 24
Phosphoglycerate mutase-like family protein	F4J8C7	4.61	333.3					0	0, 18
Phosphoinositide phosphatase SAC7	Q9C5G5	6.45	332.4	0, 12, 24	18, 24				
Phosphoinositide phospholipase C 7	Q9LY51	3.35	307.0					18	
Phospholipase D gamma 2	Q9T051	7.00	439.7					0	
Phospholipase D gamma 3	Q9T052	8.21	422.8						0
Phospholipid scramblase	Q9SI32	1.51	329.3		18, 24	12	12	0, 12, 18	0, 18
Photosystem II reaction center Psb28 protein	F4JM05	6.31	343.0				18		24
Phytochrome-associated protein phosphatase type 2C	F4I1B4	7.35	516.8			12			
PLAC8 family protein	Q8W4N1	4.32	325.9					0, 12	0, 24
PLAT domain-containing protein 1	O65660	3.34	319.8					12, 18	
**Polyubiquitin 3**	Q1EC66	10.65	611.6	24		0	12		
Porphobilinogen deaminase chloroplastic	Q43316	6.88	415.6					18	18
Probable cyclic nucleotide-gated ion channel 3	Q9SKD7	4.46	309.2					12	0, 12
Probable cytosolic oligopeptidase A	Q949P2	2.68	378.5						18
Probable fructokinase-1	Q9SID0	7.20	309.4		24	24	0		
Probable glutathione peroxidase 5	Q9LYB4	4.84	405.6					12, 18	12, 18, 24
Probable NAD(P)H dehydrogenase (quinone) FQR1-like 3	Q9LUX9	5.95	307.6	12		0, 12	0		
Probable protein phosphatase 2C 58	Q93YW5	4.81	361.6					18, 24	24
Probable protein S-acyltransferase 13	Q94C49	9.24	528.8					0, 12	0, 18

Probable sucrose-phosphate synthase 2	Q9FY54	6.07	352.5			0	0		
**Proteasome subunit alpha type-1-A**	P34066	6.97	358.0		0, 24	0, 12, 24	0, 24	18	24
**Proteasome subunit alpha type-1-B**	O23712	7.67	469.0						24
**Proteasome subunit alpha type-5-A**	O81149	8.75	465.3	24				18	
**Proteasome subunit beta type**	F4JRY2	7.36	329.2	18, 24		12		18	
**Proteasome subunit beta type-2-A**	O23714	8.47	473.0					18	
Protein CELLUL	F4IIM1	6.53	382.1			12			0
Protein disulfide isomerase-like 1-2	Q9SRG3	9.18	481.8					0, 12, 18	0, 12, 18, 24
Protein disulfide-isomerase 2-3	O48773	6.51	381.4					0, 12, 18, 24	0, 12, 18, 24
Protein DJ-1 homolog A	Q9FPF0	8.92	529.2					18	18
Protein ECERIFERUM 3	Q8H1Z0	8.20	453.7						0
Protein phosphatase 2C and cyclic nucleotide-binding/kinase domain-containing protein	Q9SL76	3.63	373.1			12	24	12	12
Protein STRICTOSIDINE SYNTHASE-LIKE 12	P94111	7.37	359.4	18	24	0, 12	0		
Protein TSS	F4JKH6	7.01	428.4	12		0, 12		0, 12	0, 18, 24
Putative lipid phosphate phosphatase 3 chloroplastic	Q8LFD1	4.93	307.6	12		12			12, 18
Pyridoxal 5'-phosphate synthase subunit PDX1.3	Q8L940	5.61	309.1					18	18
Pyruvate kinase	Q9SJQ0	6.10	337.9		24			18	0, 12, 18
RAN GTPase-activating protein 1	Q9LE82	5.91	314.6					0, 18	18
Ribose-phosphate pyrophosphokinase 4	Q680A5	9.10	466.4					24	18
S-adenosyl-L-methionine-dependent methyltransferases superfamily protein	Q8L788	8.23	413.3					0, 12, 24	0, 12
S-adenosylmethionine synthase 1	P23686	7.15	304.6	0, 24	0, 24	12, 24		18	
Serine/arginine-rich splicing factor RSZ22	O81126	7.52	411.2					12	0, 12
Serine/threonine-protein phosphatase 2A 65 kDa regulatory subunit A alpha isoform	Q38845	7.44	330.2	0, 12, 18, 24	0, 12, 18, 24			0, 18	18, 24
Serine/threonine-protein phosphatase 2A 65 kDa regulatory subunit A gamma isoform	Q38951	6.34	340.6	0, 18	0, 18, 24			0	

Signal peptidase I	Q9LW08	3.91	321.2					12	
Sorbitol dehydrogenase	Q9FJ95	8.66	569.1					0, 12, 18	0, 18, 24
Spermidine synthase 2	O48661	6.89	363.1			0, 12	0		
Staphylococcal-like nuclease CAN2	F4IH31	5.43	313.5			0			
Sterol 14-demethylase	Q9SAA9	5.17	316.8	12, 18					
Sterol 3-beta-glucosyltransferase UGT80A2	Q9M8Z7	7.27	413.1	0, 12		0, 18	0, 24	0, 12, 18	0, 12, 18, 24
Succinate--CoA ligase [ADP-forming] subunit alpha-1 mitochondrial	P68209	7.21	344.0	12, 24	18, 24				
Sucrose-phosphate synthase 1	Q94BT0	4.50	305.5	0		0, 12	0	0, 12, 18, 24	0, 12, 18
Sulfoquinovosyl transferase SQD2	Q8S4F6	6.65	393.6			0, 12, 18	0, 12, 18		
Temperature-sensitive sn-2 acyl-lipid omega-3 desaturase (ferredoxin) chloroplastic	P48622	7.74	307.6	12					
Thiamine thiazole synthase chloroplastic	Q38814	6.90	404.6			0	18	18	18
Thioredoxin-like protein HCF164 chloroplastic	O23166	7.77	373.8	24	12, 18				18
Tropinone reductase homolog At5g06060	Q9LHT0	4.23	352.0			12	24		18
**Ubiquitin-activating enzyme E1 1**	P93028	7.45	381.2					12	0, 18
**Ubiquitin-conjugating enzyme 36**	F4I615	7.31	336.9		18, 24				
**Ubiquitin-NEDD8-like protein RUB1**	Q9SHE7	9.01	450.9	12, 24	0	0, 18	12, 18		
U-box domain-containing protein 44	Q9LM76	5.98	304.3				0, 18, 24	0, 12	0, 12, 18
Xyloglucan glycosyltransferase 4	Q9LJP4	3.89	307.1						0
**Perception and Signalling**									
14-3-3-like protein GF14 epsilon	P48347	8.62	404.6					0, 18	0, 12, 18, 24
14-3-3-like protein GF14 upsilon	P42645	6.75	417.0					12, 24	0, 24
Adenosine kinase 2	Q9LZG0	9.20	471.7					24	
At4g32250/F10M6_110	Q8RWX4	4.82	366.8	12		12			
AT5G05670 protein	Q9FFK7	7.15	322.7			0, 12, 18	12	0, 18, 24	0, 12, 18
ATMRK1	O22100	4.18	310.0		18	12, 18			
Auxin-induced in root cultures protein 12	Q94BT2	6.52	308.8	18, 24	18, 24	0, 12, 18, 24	0, 12, 18, 24	18	18
Calcium-binding EF hand family protein	Q9FNN0	6.24	339.0					12, 18	0
**Calcium-dependent protein kinase 13**	Q8W4I7	4.55	325.9					0	0

**Calcium-dependent protein kinase 29**	Q8RWL2	6.09	339.2					0, 12	0, 12
**Calcium-dependent protein kinase 3**	Q42479	7.76	409.6			0, 12, 18, 24	0, 12, 18, 24		
**Calcium-dependent protein kinase 32**	Q6NLQ6	7.75	307.7	0, 12, 18, 24	18, 24				
**Calcium-dependent protein kinase 33**	Q9C6P3	7.95	313.7		12				
**Calcium-dependent protein kinase 5**	Q38871	6.82	301.2	12		12, 24	24	0	0, 12, 18, 24
**Calcium-dependent protein kinase 7**	Q38873	4.59	310.2	12		0			
**Calcium-dependent protein kinase 8**	Q42438	4.59	310.2	12		0			
**Calcium-dependent protein kinase 9**	Q38868	7.80	307.5	12, 24	18, 24	12	0, 18, 24		
CBL-interacting serine/threonine-protein kinase 26	Q84VQ3	6.89	409.4					0	18
CDPK-related kinase 1	O80673	7.51	347.1						12
**Chitin elicitor receptor kinase 1 (CERK1)**	A8R7E6	4.67	311.0				0, 24	0, 12	0, 12, 18, 24
**Cysteine-rich receptor-like protein kinase 17**	Q8L710	4.46	360.2	18	0, 12, 18, 24	0, 12, 24	0, 24	12	0, 12
**Cysteine-rich receptor-like protein kinase 18**	Q8RX80	4.97	372.7				0		
**Cysteine-rich receptor-like protein kinase 19**	Q8GWJ7	3.87	357.4			18			
**Cysteine-rich receptor-like protein kinase 2**	Q9CAL3	6.17	339.9	12	18	12	0, 18, 24		
**Cysteine-rich receptor-like protein kinase 21**	Q3E9X6	4.99	327.4						0, 12
**Cysteine-rich receptor-like protein kinase 3**	Q9CAL2	8.29	455.9						0
**Cysteine-rich receptor-like protein kinase 4**	Q9LZU4	3.87	357.4			18			
**Cysteine-rich receptor-like protein kinase 41**	O23081	4.59	365.2					0	0
**Cysteine-rich receptor-like protein kinase 6**	Q9C5S9	6.77	361.6					18	12
**Cysteine-rich receptor-like protein kinase 8**	O65468	6.77	361.6					18	12
Diacylglycerol kinase 3	Q8VZG1	7.41	303.4			24	12, 18, 24		
Diacylglycerol kinase 7	F4JQ95	7.96	361.8		18	0	0		
ERBB-3 BINDING PROTEIN 1	Q96327	7.44	415.0			0, 12, 24	12, 24	18	18
GF14 protein phi chain	F4HWQ5	8.58	546.3					18	18
**G-type lectin S-receptor-like serine/threonine-protein kinase At1g61440**	O64776	2.43	306.9						18

**G-type lectin S-receptor-like serine/threonine-protein kinase At2g19130**	O64477	2.72	306.8			0, 12			
**G-type lectin S-receptor-like serine/threonine-protein kinase At4g03230**	Q9ZR08	5.05	407.7						12
G-type lectin S-receptor-like serine/threonine-protein kinase SD1-29	O64782	6.52	361.9			24			
Histidine kinase 3	Q9C5U1	3.65	324.9					18	0, 18
**Inactive LRR receptor-like serine/threonine-protein kinase BIR2**	Q9LSI9	7.22	471.6			12, 18			
Inositol hexakisphosphate and diphosphoinositol-pentakisphosphate kinase VIP1	F4J8C6	4.61	333.3					0	0, 18
Integrin-linked protein kinase family	F4J703	5.96	346.7					12	0
Kinase like protein	O23334	6.07	313.9		0	0			
Kinase with tetratricopeptide repeat domain-containing protein	A8MQ88	5.29	355.0	12					12
LEAF RUST 10 DISEASE-RESISTANCE LOCUS RECEPTOR-LIKE PROTEIN KINASE-like 1.4	F4HQ17	5.14	371.3						0
Leucine-rich repeat protein kinase family protein	F4IB69	3.94	346.6				18, 24		18
Leucine-rich repeat protein kinase family protein (Fragment)	C0LGM1	6.12	332.2		12	12		12, 18	0, 12, 18, 24
**Leucine-rich repeat receptor-like serine/threonine/tyrosine-protein kinase SOBIR1**	Q9SKB2	8.17	409.1	12	0				
Leucine-rich repeat receptor-like serine/threonine-protein kinase BAM1	O49545	7.05	501.0	0, 12, 18, 24	0, 12, 18, 24				
Long-chain base (LCB) kinase 1	B3H501	9.60	459.8					18, 24	
LRR receptor-like serine/threonine-protein kinase GSO2	Q9FIZ3	1.48	329.6			12	12		
LRR receptor-like serine/threonine-protein kinase HSL2	C0LGX3	2.90	387.8				24		

**L-type lectin-domain containing receptor kinase IV.3**	O81292	1.93	308.0				0		
**L-type lectin-domain containing receptor kinase IV.4**	O81291	5.34	359.3					0, 12, 18	0, 12, 24
**L-type lectin-domain containing receptor kinase S.1**	Q9M9E0	4.03	317.7		24	12		12	
**L-type lectin-domain containing receptor kinase VII.1**	Q9S9U1	3.53	313.8	18					
**LysM domain receptor-like kinase 3 (LYK3)**	F4IB81	5.03	306.2				24	0, 12	
**LysM domain receptor-like kinase 4 (LYK4)**	O64825	7.20	309.8					18	
MAP3K epsilon protein kinase 1	Q9LJD8	7.37	404.9	12, 18, 24	12, 18, 24			0, 12	0
Mitogen-activated protein kinase kinase 2	Q9S7U9	6.81	303.7	0	0	18			
Phosphatidylinositol 4-kinase alpha 1	Q9SXA1	7.07	319.7			12			
Phosphoinositide phospholipase C	F4IX90	13.43	717.2					18	
Phosphoinositide phospholipase C 1	Q39032	7.46	361.1		12				
Probable calcium-binding protein CML20	O82659	7.09	359.3	0, 12, 18, 24	0, 12	12, 24			
Probable inactive receptor kinase At1g27190 BIR3	O04567	7.37	358.9			12, 18, 24	0, 18	0, 12, 18	0, 12, 18, 24
Probable inactive receptor kinase At3g08680	Q9C9Y8	6.93	354.3	0					
Probable inactive receptor kinase At5g10020	Q0WR59	1.88	342.3					18	18
Probable inactive receptor kinase RLK902	Q9LVI6	8.12	409.3	12, 18, 24	18, 24			18	18
Probable LRR receptor-like serine/threonine-protein kinase At1g06840	C0LGD7	7.72	336.7	24	18				
Probable LRR receptor-like serine/threonine-protein kinase At1g12460	C0LGE4	1.70	314.7				18		
Probable LRR receptor-like serine/threonine-protein kinase At1g53430	C0LGG8	6.98	444.5	24	0, 12, 24			0, 12, 18	12, 18, 24
Probable LRR receptor-like serine/threonine-protein kinase At1g53440	C0LGG9	7.57	434.1					0, 12, 18	0

Probable LRR receptor-like serine/threonine-protein kinase At1g56140	C0LGH3	2.58	381.3			0		12, 18	12
Probable receptor-like protein kinase At1g30570	Q9SA72	5.90	301.2	18					
Probable receptor-like protein kinase At1g49730	Q9FX99	6.64	328.2	18	24	12			12
Probable receptor-like protein kinase At2g23200	O22187	7.69	311.9	12					
Probable receptor-like protein kinase At5g18500	Q8LEB6	6.28	304.1	24	18, 24	0	0, 24		
Probable receptor-like protein kinase At5g38990	Q9FID9	8.16	399.5	12	18, 24	0, 18, 24	0, 24	0, 12, 18	0, 18, 24
Probable serine/threonine protein kinase IREH1	F4J6F6	6.99	392.1					12	
Probable serine/threonine-protein kinase At1g54610	Q9ZVM9	8.62	407.6					0	0
Probable serine/threonine-protein kinase CST	P27450	8.35	448.2					0, 12	0, 12
**Probable serine/threonine-protein kinase PBL1**	Q8H186	6.68	337.0						18
**Probable serine/threonine-protein kinase PBL11**	P43293	5.94	300.4				0, 24	0	0
**Probable serine/threonine-protein kinase PBL2**	O49839	3.26	346.8			0			
**Probable serine/threonine-protein kinase PBL22**	Q9SFX0	3.90	354.8			12	24		
**Probable serine/threonine-protein kinase PBL24**	Q1PE89	3.83	346.1				12		
**Probable serine/threonine-protein kinase PBL3**	O49840	3.26	346.8			0			
**Probable serine/threonine-protein kinase PBL7**	Q0WRY5	3.20	347.4				18		
Probably inactive leucine-rich repeat receptor-like protein kinase At3g28040	Q9LRT1	7.17	325.0		18			18	0, 12, 18
**Probably inactive leucine-rich repeat receptor-like protein kinase BIR1 At5g48380**	Q9ASS4	7.73	465.4			18	18, 24		
Proline-rich receptor-like protein kinase PERK2	Q9LK03	2.35	329.1				0		
Proline-rich receptor-like protein kinase PERK3	Q1PEM5	9.02	389.4	12					
Protein BRASSINOSTEROID INSENSITIVE 1	O22476	7.31	340.1					12	0
Protein kinase	Q9FGB1	2.92	301.6	12, 18, 24	24	12	24		0
Protein kinase superfamily protein At1g48210	F4HWU0	8.70	559.9			0, 12, 18, 24	0, 18, 24	0	0, 12, 18

Protein kinase superfamily protein At1g52540	Q8LDB7	7.41	379.9	12, 18	0	12	0		
Protein kinase superfamily protein At1g65190	Q9S9J9	4.27	366.6					12	12
Protein kinase superfamily protein At3g26700	F4JDN8	5.17	385.8		18			0, 12, 18	0, 24
Protein kinase superfamily protein At4g01330	F4JI10	3.48	321.3		18				
Protein kinase superfamily protein At5g14720	Q84WU5	4.94	306.6						0
Protein kinase superfamily protein Atg49730	F4I3D1	6.64	328.2	18	24	12			12
Protein STRUBBELIG-RECEPTOR FAMILY 6	Q9C8M9	5.38	346.7					0, 18	18
PTI1-like tyrosine-protein kinase 1	Q8H1G6	7.66	372.1	18		0, 12, 24	0, 24		
PTI1-like tyrosine-protein kinase 2	O49339	7.07	373.9		0, 12, 18			0, 12, 18	0, 12, 18, 24
PTI1-like tyrosine-protein kinase At3g15890	Q9LSC2	7.41	379.9	12, 18	0	12	0		
**Putative cysteine-rich receptor-like protein kinase 12**	O65472	4.21	344.3	24					
**Putative cysteine-rich receptor-like protein kinase 30**	Q9LDT0	1.52	344.1	12	12, 18		0, 18		
**Putative cysteine-rich receptor-like protein kinase 31**	Q9LDM5	1.52	344.1	12	12, 18				
Putative leucine-rich repeat receptor-like serine/threonine-protein kinase At2g04300	Q9SI06	2.11	307.1						0
Putative proline-rich receptor-like protein kinase PERK6	Q9LS95	3.02	345.7		24				
Putative receptor-like protein kinase At5g39000	Q9FID8	8.16	399.5	12					
Receptor protein kinase TMK1	P43298	7.31	334.4			0, 12		0, 12, 18, 24	0, 12, 18, 24
**Receptor-like protein 51 RLP51**	Q9SN38	7.39	359.0					18	18
Receptor-like protein kinase At5g59670	Q9FN94	6.44	395.1						0, 24
Receptor-like protein kinase HERK 1	Q9LX66	7.31	377.8					0, 12, 18, 24	0, 12, 18, 24
Receptor-like protein kinase HSL1	Q9SGP2	4.99	305.9	12	18, 24	0, 12			18
Receptor-like protein kinase THESEUS 1	Q9LK35	7.37	321.8	0, 18					

Receptor-like serine/threonine-protein kinase SD1-7	Q39086	5.79	395.7						0
**Serine/threonine-protein kinase BSK2**	Q9LS26	3.03	315.8	24		0			18
**Serine/threonine-protein kinase BSK3**	Q8W4L3	3.50	315.0		18, 24				
**Serine/threonine-protein kinase BSK5**	Q9FIL1	5.29	355.0	12				0	12
**Serine/threonine-protein kinase BSK6**	Q9M324	6.18	310.8					12	
**Serine/threonine-protein kinase BSK8**	Q9FHD7	4.48	323.2		12		0		
Serine/threonine-protein kinase CDG1	Q9LSE1	4.08	342.0	24					
**Serine/threonine-protein kinase PBL27**	Q1PDV6	3.79	359.4	18	18				
Serine/threonine-protein kinase PBS1	Q9FE20	3.77	351.4				0		
**Serine/threonine-protein kinase RIPK**	Q9ZUF4	4.08	341.4	0, 12	0, 12, 24	18, 24			
Serine/threonine-protein phosphatase 2A 65 kDa regulatory subunit A beta isoform	Q38950	8.50	450.9						0
Signal recognition particle 54 kDa protein chloroplastic	P37107	7.11	452.3					18	
**Somatic embryogenesis receptor kinase 2 (SERK2)**	Q9XIC7	7.93	341.8		12				
**Somatic embryogenesis receptor kinase 4 (SERK4)/BKK1**	Q9SKG5	5.73	338.5					0, 12, 18	0
STRUBBELIG-receptor family 6	A8MQH3	7.96	470.2					0, 18	0, 18
Wall-associated receptor kinase 2	Q9LMP1	7.16	381.4	12, 18, 24		12	0, 12	12	12
Wall-associated receptor kinase 4	Q9LMN6	7.15	372.4		12, 18				
Wall-associated receptor kinase 5	Q9LMN7	9.31	539.8					12	0, 12, 24
**Stress and Defence Response**									
2-Cys peroxiredoxin BAS1-like chloroplastic	Q9C5R8	7.02	567.2			0, 12, 24	18, 24		
Ankyrin repeat-containing protein 2	F4JN13	6.95	329.8	12, 18, 24	0	12	0		
At2g44060	O80576	6.96	538.6					0, 12, 18	0, 18, 24
AT4g34150/F28A23_90	Q945K9	6.89	359.6	12, 24	12	0, 12, 24	0, 12, 24		
AT4g34180/F28A23_60	Q93V74	9.13	452.9					18	

BAG family molecular chaperone regulator 8 chloroplastic	Q9LIB3	6.95	395.0					0	0, 12
Binding partner of ACD11 1	Q9LFD5	6.26	420.0	0, 12	0, 12, 18, 24	0, 12, 18, 24	0, 12, 18, 24		
Chaperone protein dnaJ 2	P42825	5.53	332.8				18		
Chaperone protein dnaJ 3	Q94AW8	5.36	363.8			0, 12	0, 18		
Chaperonin 60 subunit beta 1 chloroplastic	P21240	7.10	498.4					0, 18, 24	0, 12, 18, 24
Cold shock protein 2	Q41188	7.84	306.8	24	0		0		
Cold-regulated 413 plasma membrane protein 1	Q9XIM7	6.27	375.1					0	12, 18, 24
Cold-responsive protein kinase 1	Q93YN1	6.59	401.8					0	0
Cysteine proteinase inhibitor 1	Q945Q1	8.76	533.9	0, 12, 18, 24	0, 12, 18	12, 18			
DCD (Development and Cell Death) domain protein	F4K518	4.66	363.8					0	12
DEAD-box ATP-dependent RNA helicase 38	Q93ZG7	7.97	400.1	12, 18				18	18
Derlin-1	Q8VZU9	4.30	309.2	0				12	0, 12
**Disease resistance protein RPP8**	Q8W4J9	5.53	370.9	12		0, 12, 18, 24	0, 12, 18, 24		
**Disease resistance RPP13-like protein 4**	Q38834	7.03	479.9					0, 12	0, 12
DnaJ protein P58IPK homolog	Q9LYW9	6.39	461.6					0, 12	0, 12
Glutathione S-transferase DHAR1 mitochondrial	Q9FWR4	5.05	380.8					18	
Glutathione S-transferase U5	P46421	6.30	370.4					18	
Guanine nucleotide-binding protein subunit beta	P49177	4.34	312.2				24		0
**Heat shock 70 kDa protein 14**	Q9S7C0	5.02	337.1	12, 18	18				
**Heat shock 70 kDa protein 3**	O65719	7.48	427.5			12, 18, 24	0, 18, 24		
**Heat shock protein 90-2**	P55737	5.24	370.2					24	0, 12, 18, 24
**Heat shock protein 90-3**	P51818	7.04	378.0	0	24	24	0, 12, 18, 24		
Hypersensitive-induced response protein 1	Q9FM19	6.33	420.1					0, 12, 18, 24	0, 12, 18, 24
LRR and NB-ARC domains-containing disease resistance protein	Q2V4G0	3.71	366.9						0
Metacaspase-4	O64517	7.29	334.9				24		
MLO-like protein 1	O49621	8.65	460.9					18	
MLO-like protein 2	Q9SXB6	8.76	461.4					0, 12, 24	0, 12
Nucleophosmin	Q9SU93	6.72	425.5	0			12	0, 18, 24	0, 18
Pectinesterase/pectinesterase inhibitor 3	O49006	6.67	396.3	18, 24	24	12, 24	0, 12, 24		
Phospholipase D gamma 1	Q9T053	7.95	412.6					12	0, 12

**Probable disease resistance RPP8-like protein 4**	Q9FJK8	5.75	306.2		18, 24				
Probable glutathione peroxidase 2	O04922	6.53	332.5	24					
Probable phospholipid hydroperoxide glutathione peroxidase 6 mitochondrial	O48646	7.11	443.2	0, 18, 24	0, 12, 18, 24			18	
Proline-rich receptor-like protein kinase PERK1	Q9LV48	5.25	336.3	18		12	0	0, 12, 24	0, 12, 18, 24
Protein BONZAI 1	Q941L3	4.66	363.8					0	12
Protein EARLY-RESPONSIVE TO DEHYDRATION 7 chloroplastic	O48832	9.01	446.5	12, 18					
**Protein SUPPRESSOR OF NPR1-1 CONSTITUTIVE 4**	D7SFH9	3.60	332.8	12		0		0, 18, 24	0, 12
Protein-tyrosine-phosphatase PTP1	O82656	4.98	310.2			0			
**Putative disease resistance protein RLP23**	O48849	7.38	307.1			12, 24	24	12	0, 12
**Putative respiratory burst oxidase homolog protein J (RbohJ)**	Q9LZU9	1.53	321.7		12				
**Respiratory burst oxidase homolog protein D (RbohD)**	Q9FIJ0	1.53	321.7		12		0	0, 12, 18	0, 12, 18, 24
Ribonuclease TUDOR 1	Q8VZG7	5.61	349.8	0				0, 12, 18	18
RNA-binding (RRM/RBD/RNP motifs) family protein	F4HVJ0	7.18	445.7					12	12
RPM1-interacting protein 4	Q8GYN5	7.18	316.7			0, 12			
Superoxide dismutase [Fe] 1 chloroplastic	P21276	7.80	443.5						0
Tetraspanin-8	Q8S8Q6	5.71	361.5					0	
Thioredoxin H3	Q42403	6.82	389.8					0, 18, 24	0, 18
Thioredoxin H5	Q39241	4.79	337.4			24			
Universal stress protein PHOS32	Q8VYN9	5.03	341.9	18, 24					

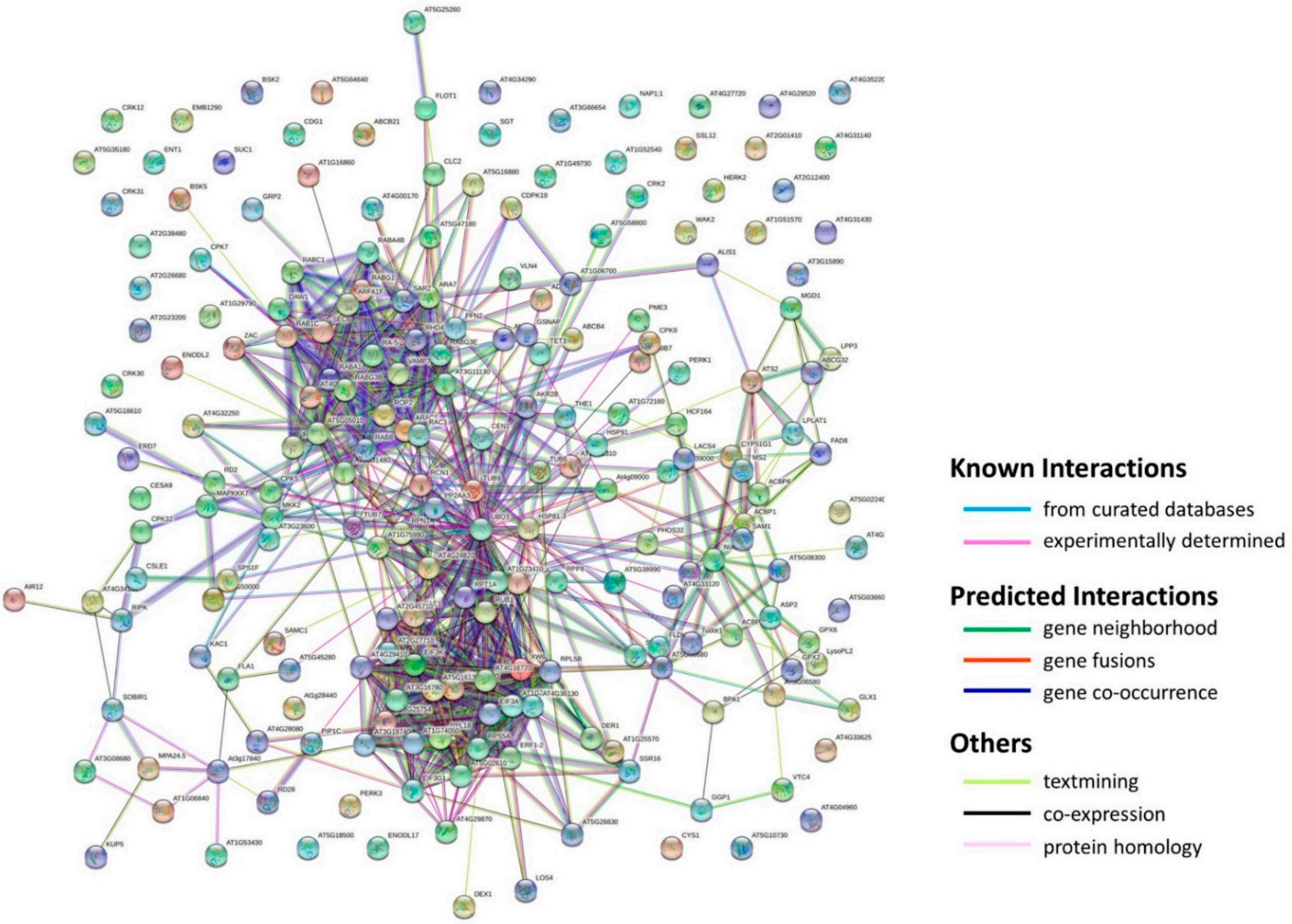

Figure S3. The protein–protein interaction networks of PM-associated proteins identified from LPS$_{Pst}$-treated Arabidopsis WT. For noting and clarity, the clustering differences are presented for significant differences and not specific proteins in small font. Circles: significant proteins; Straight lines: the interactions between different proteins; Colours: type of evidence for connection.

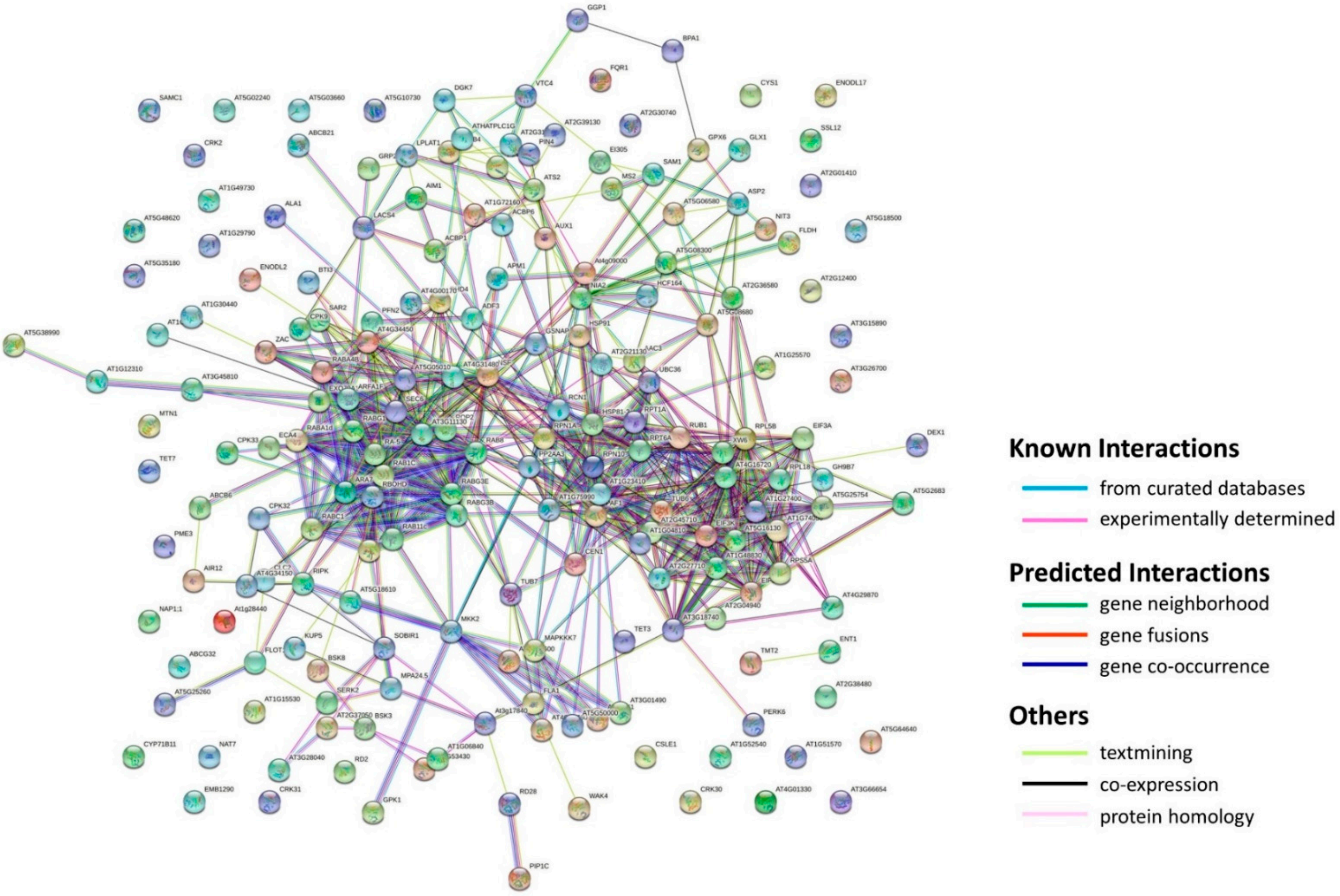

Figure S4. The protein–protein interaction networks of PM-associated proteins identified from LPS$_{Xcc}$-treated Arabidopsis WT. Circles: significant proteins; Straight lines: the interactions between different proteins; Colours: type of evidence for connection.

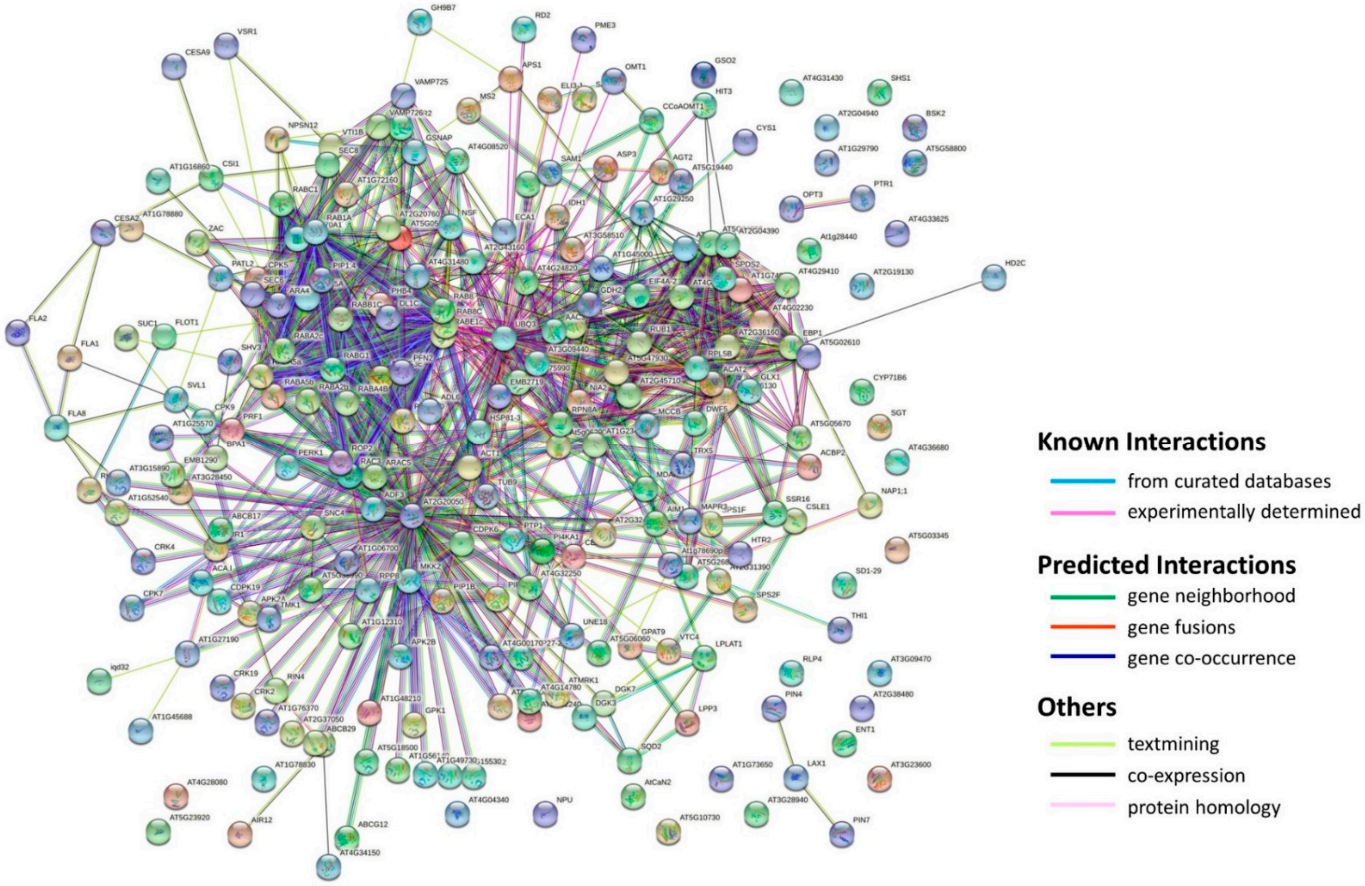

Figure S5. The protein–protein interaction networks of PM-associated proteins identified from LPS$_{Pst}$-treated Arabidopsis *lbr2-2*. Circles: significant proteins; Straight lines: the interactions between different proteins; Colours: type of evidence for connection.

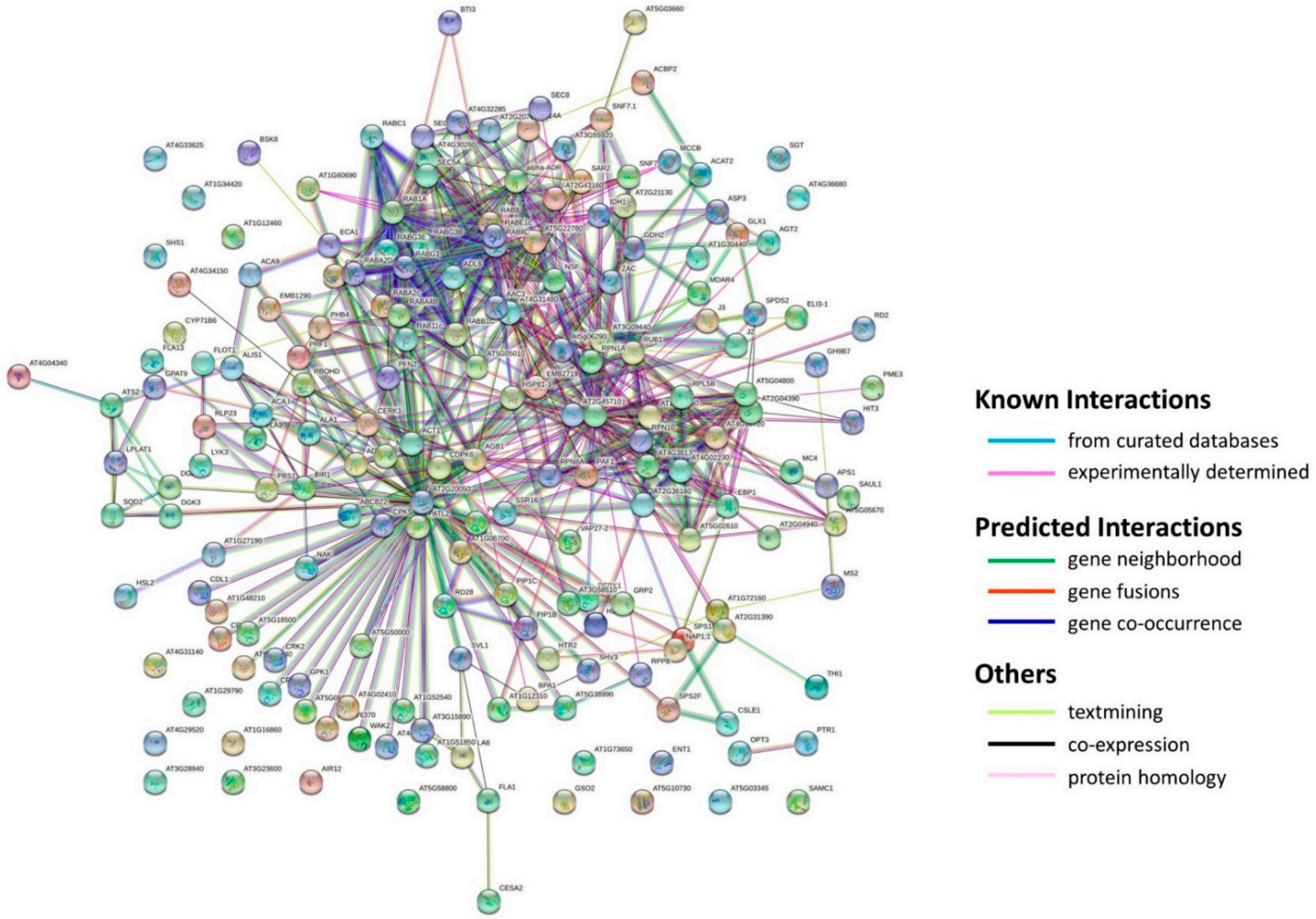

Figure S6. The protein–protein interaction networks of PM-associated proteins identified from LPS$_{Xcc}$-treated Arabidopsis *lbr2-2*. Circles: significant proteins; Straight lines: the interactions between different proteins; Colours: type of evidence for connection.

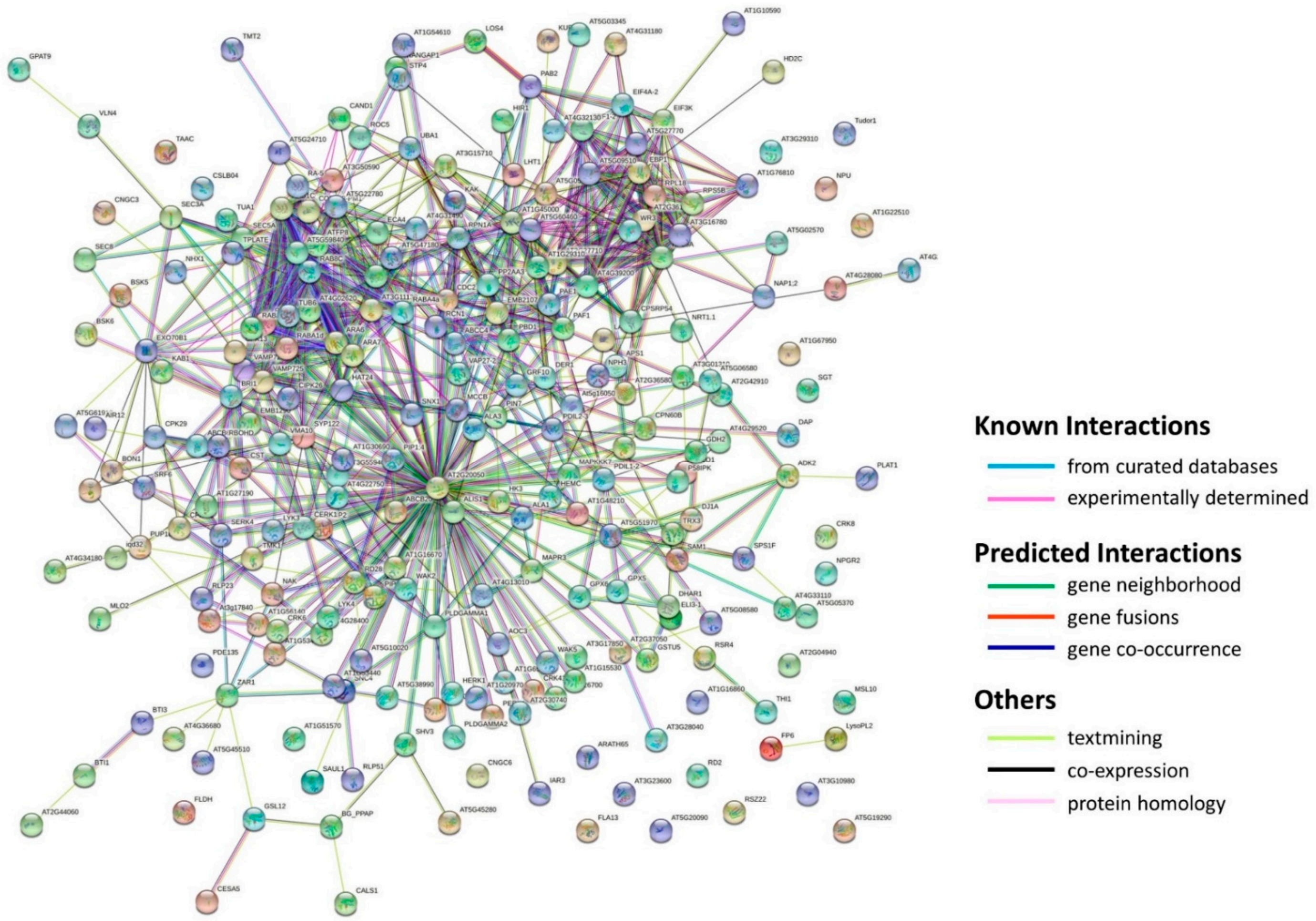

Figure S7. The protein–protein interaction networks of PM-associated proteins identified from LPS$_{Pst}$-treated Arabidopsis *bak1-4*. Circles: significant proteins; Straight lines: the interactions between different proteins; Colours: type of evidence for connection.

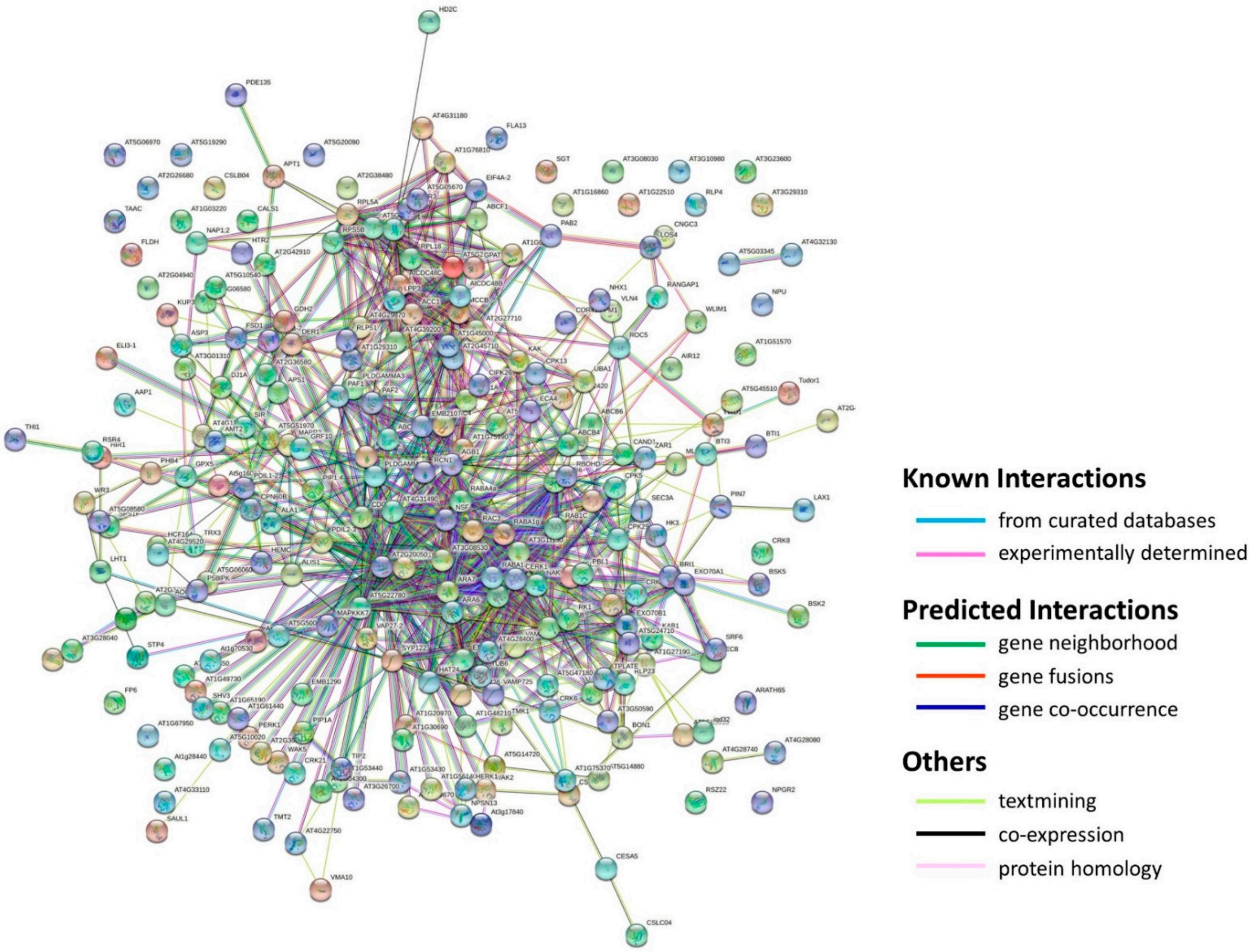

Figure S8. The protein–protein interaction networks of PM-associated proteins identified from LPS_{Xcc}-treated Arabidopsis *bak1-4*. Circles: significant proteins; Straight lines: the interactions between different proteins; Colours: type of evidence for connection.

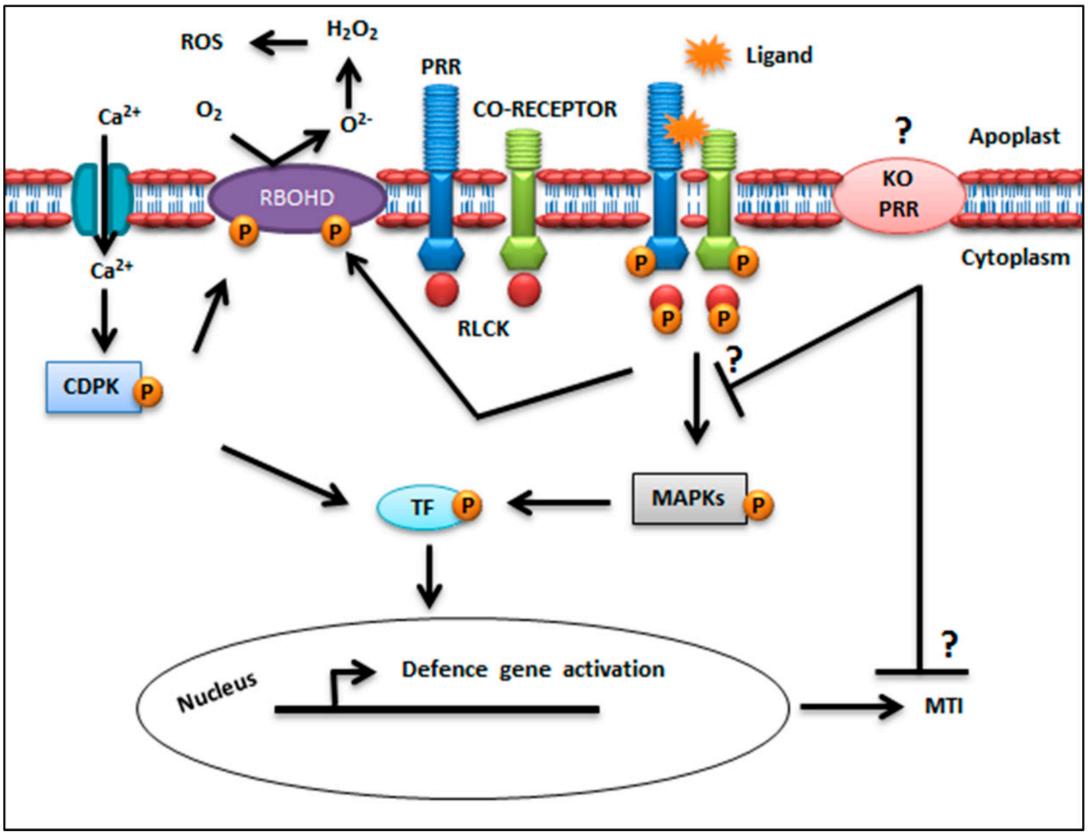

Figure S9. Generalised view of MAMP perception via PRRs, illustrating the potential impact of knockout (KO) effects that influence MAMP-triggered immunity. Upon MAMP (e.g., flg22) perception by a cognate PRR (e.g., in the case of FLS2 [**39**], the RLCK Botrytis induced kinase1 (BIK1) dissociates from its complex with FLS2 and co-receptor BAK1. This leads to the activation of defence responses such as ROS production, cytoplasmic Ca^{2+} influx, activation of downstream signalling kinases (MAPK and CDPK) and upregulation of defence genes. KO of the PRR and/or co-receptor (demarcated by the dotted line) may impair activation of downstream defence signalling, that could possibly lead to increased susceptibility of a plant to a pathogen. P = Phosphorylation, RBOHD = Respiratory burst oxidase homolog D, TF = Transcription factor, PRR = Pattern recognition receptor, CR = Co-receptor, MAMP = Microbe-associated molecular pattern, ROS = Reactive oxygen species, CDPK = Calcium-dependent protein kinase, MAPK = Mitogen-activated protein kinase, RLCK = Receptor-like cytoplasmic kinase, MTI = MAMP-triggered immunity (taken from [**2**]).

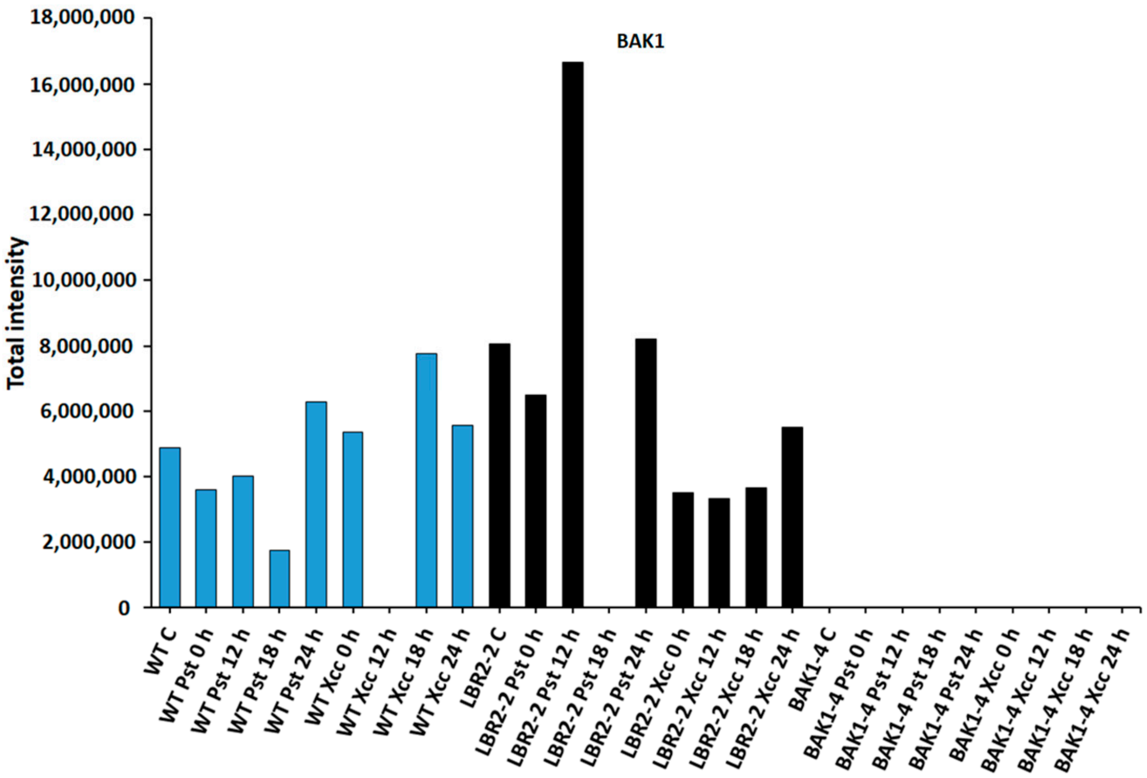

Figure S10. Analysis of total peak intensity of PM-associated BAK1 detected in the control, LPS$_{Pst}$ - and LPS$_{Xcc}$-treated WT and *lbr2-2* within a 24 h period. Even though BAK1 was detected above the significance threshold scores in both LPS$_{Pst}$ and LPS$_{Xcc}$-treated WT and *lbr2-2*, it was not included in the discussion due to detection within the significance threshold scores of the $MgCl_2$-control at the 24 h period post-treatment.
